# Enhanced TRPC3 transcription through AT1R/PKA/CREB signaling contributes to mitochondrial dysfunction in renal tubular epithelial cells in D‐galactose‐induced accelerated aging mice

**DOI:** 10.1111/acel.14130

**Published:** 2024-02-28

**Authors:** Bin Wang, Wenpei Yu, Weiguang Zhang, Min Zhang, Yue Niu, Xinye Jin, Jie Zhang, Ding Sun, Hao Li, Zehao Zhang, Qing Luo, Xiaowei Cheng, Jingxue Niu, Guangyan Cai, Xiangmei Chen, Yizhi Chen

**Affiliations:** ^1^ Department of Nephrology, The Hainan Academician Team Innovation Center Hainan Hospital of Chinese PLA General Hospital Sanya China; ^2^ Senior Department of Nephrology, The First Medical Center of Chinese PLA General Hospital Chinese PLA Institute of Nephrology, State Key Laboratory of Kidney Diseases, National Clinical Research Center for Kidney Diseases, Beijing Key Laboratory of Kidney Diseases Research Beijing China; ^3^ Department of Chemical Defense Medicine, School of Military Preventive Medicine Army Medical University (Third Military Medical University) Chongqing China; ^4^ Department of Clinical Medicine Dazhou Vocational and Technical College Dazhou Sichuan China; ^5^ Graduate School Chinese PLA General Hospital Beijing China; ^6^ The Second School of Clinical Medicine Southern Medical University Guangzhou China

**Keywords:** cell senescence, cyclic AMP response element‐binding protein, kidney tubules, mitochondrial disorders, transient receptor potential cation channel, subfamily C, member 3

## Abstract

Aging‐associated renal dysfunction promotes the pathogenesis of chronic kidney disease. Mitochondrial dysfunction in renal tubular epithelial cells is a hallmark of senescence and leads to accelerated progression of renal disorders. Dysregulated calcium profiles in mitochondria contribute to aging‐associated disorders, but the detailed mechanism of this process is not clear. In this study, modulation of the sirtuin 1/angiotensin II type 1 receptor (Sirt1/AT1R) pathway partially attenuated renal glomerular sclerosis, tubular atrophy, and interstitial fibrosis in D‐galactose (D‐gal)‐induced accelerated aging mice. Moreover, modulation of the Sirt1/AT1R pathway improved mitochondrial dysfunction induced by D‐gal treatment. Transient receptor potential channel, subtype C, member 3 (TRPC3) upregulation mediated dysregulated cellular and mitochondrial calcium homeostasis during aging. Furthermore, knockdown or knockout (KO) of *Trpc3* in mice ameliorated D‐gal‐induced mitochondrial reactive oxygen species production, membrane potential deterioration, and energy metabolism disorder. Mechanistically, activation of the AT1R/PKA pathway promoted CREB phosphorylation and nucleation of CRE2 binding to the *Trpc3* promoter (−1659 to −1648 bp) to enhance transcription. *Trpc3* KO significantly improved the renal disorder and cell senescence in D‐gal‐induced mice. Taken together, these results indicate that TRPC3 upregulation mediates age‐related renal disorder and is associated with mitochondrial calcium overload and dysfunction. TRPC3 is a promising therapeutic target for aging‐associated renal disorders.

## Graphical abstract

During aging, dysregulated Sirt1/AT1R/CREB signaling promotes TRPC3 transcription in renal tubular epithelial cells. TRPC3 upregulation mediates mitochondrial calcium overload, mitochondrial redox imbalance, and respiratory dysfunction, leading to accelerated renal aging. TRPC3 deficiency partially ameliorates aging‐associated renal disorders and is a potential therapeutic target.

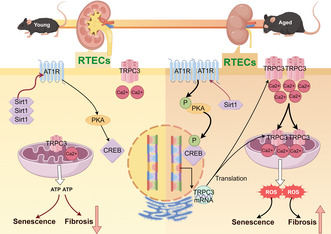

**Abbreviations**
AT1R: angiotensin II type 1 receptor
ChIP: chromatin immunoprecipitation
CKD: chronic kidney disease
CREB: cAMP‐response element binding protein
D‐gal: D‐galactose
DHE: dihydroethidium
FBS: fetal bovine serum
GAPDH: glyceraldehyde‐3‐phosphate dehydrogenase
H&E: hematoxylin and eosin
MMP: mitochondrial membrane potential
Nrf2: nuclear factor erythroid 2‐related factor 2
OAG: oleyl‐acetyl glycerol
PAS: periodic acid‐Schiff
PKA: protein kinase A
RAS: renin‐angiotensin system
ROS: reactive oxygen species
RTECs: renal tubular epithelial cells
SA‐β‐gal: senescence‐associated β‐galactosidase activity
Sirt1: sirtuin 1
SOCE: store‐operated calcium entry
STAT‐3: signal transducer and activator of transcription‐3
TG: Thapsigargin
TRPC3: transient receptor potential channel, subtype C, member 3
UACR: urinary albumin to urinary creatinine ratio

## 1 INTRODUCTION

Aging is a huge health burden worldwide, with kidneys being particularly susceptible to aging‐associated changes (O'Sullivan et al., 2017). Aging kidneys are vulnerable to both intrinsic and environmental challenges, which result in the increased incidence of chronic kidney disease (CKD) with age. Aged individuals over 70 years old are at risk of a 13‐fold increase in CKD morbidity compared with young subjects (Minutolo et al., 2015). Aging‐associated renal changes include glomerulosclerosis, arteriosclerosis, tubular atrophy and interstitial fibrosis, while cell senescence plays an important role in renal aging (Fang et al., 2020). Therefore, investigating the mechanism of cell senescence may provide more clues and potential targets for CKD treatments.

Among various animal models of aging, D‐galactose (D‐gal)‐induced models have been widely used to investigate aging mechanisms because of their convenience, lack of side effects, and a high survival rate throughout the experimental period (Azman & Zakaria, 2019). D‐gal is an aldohexose, a reducing sugar that exists naturally in the body and in many foods. Chronic administration of D‐gal may enhance the generation of reactive oxygen species (ROS), causing physiological states similar to those observed during natural aging, such as cell metabolism disorders and injury (Wang et al., 2022). More importantly, D‐gal‐induced aging models are widely used in the study of renal aging because they mimic the aging process, with increased oxidative stress, lipid hydroperoxide levels, inflammatory status, and impaired renal functions (Azman & Zakaria, 2019). Renal tubules account for more than 90% of the total renal mass and are the kidney structures most affected by aging (Fang et al., 2020). Renal tubular epithelial cells (RTECs) represent the major type of kidney parenchymal cell and play dominate roles in reabsorption and secretion of nutrients, water, and key ions. Therefore, a high energy demand and large number of mitochondria are key characteristics of RTECs. Mitochondria have essential roles in both energy production and redox balance; therefore, their normal functions are important for cellular homeostasis (Miao et al., 2019). Mitochondrial dysfunction‐induced impaired energy production and excessive oxidative stress are the major contributors to aging‐associated pathologies (Uzhachenko et al., 2017). Furthermore, mitochondrial dysfunction of RTECs contributes to a variety of CKDs, with increased ROS generation and activation of inflammation, leading to accelerated progression of renal fibrosis (Miguel et al., 2021). These findings highlight the importance of identifying the mechanisms that underlie mitochondrial dysfunction in RTECs for understanding aging‐associated renal disorders (Lee et al., 2021).

Among the various regulatory functions of mitochondria, calcium homeostasis is considered important in various pathological conditions. Dysregulated calcium status exists in aged mesenchymal stem cells and aging rat aorta (Ahamad et al., 2021; Erac et al., 2010), while we have previously shown that modulation of mitochondrial calcium overload ameliorates age‐associated disorders (Gao et al., 2020). Moreover, calcium, ROS, and mitochondria comprise a close triangle that modulate not only cell function and cell fate, but also functions involved in aging and age‐associated diseases (Madreiter‐Sokolowski et al., 2020). Among the various calcium channels, transient receptor potential canonical (TRPC) channels are a group of nonselective cation channels that are widely expressed and exhibit a close relationship with mitochondrial metabolism (Nan et al., 2021). Generally, TRPC channels control the flux of cations across the plasma membrane and are permeable to both monovalent cations (Na^+^ and K^+^) and divalent cations (Ca^2+^ and Mg^2+^). Among them, transient receptor potential channel, subtype C, member 3 (TRPC3) (TRP, subtype C, member 3) has been considered to be localized in the plasma membrane; however, recent study by Feng et al. (2013) and ourselves (Wang et al., 2017) show that TRPC3 may also be localized in mitochondria. Moreover, a fraction of TRPC3‐interacting proteins investigated by mass spectrometry was associated with mitochondria (Lockwich et al., 2008). Therefore, TRPC3 might be involved with mitochondrial metabolism. Electrophysiologically, TRPC3 channels are more permeable to Na^+^ than to Ca^2+^ ions (P_Na_: P_Ca_~6:1) (Earley & Brayden, 2015). TRPC3‐mediated Na^+^ influx may increase intracellular Ca^2+^ levels by activating voltage‐dependent Ca^2+^ channels or the Na^+^/Ca^2+^ exchanger. We have demonstrated that enhanced cellular and mitochondrial expression of TRPC3 in hypertension modulates mitochondrial calcium entry and respiratory functions (Wang et al., 2017). Moreover, TRPC3 is upregulated in atrial fibrosis in both aging and spontaneously hypertensive rats (He et al., 2019). However, it is unknown whether mitochondrial dysfunction in aging RTECs correlates with TRPC3 and investigating TRPC3‐associated mitochondrial dysfunction in RTECs may help reveal the mechanisms of RTEC senescence and renal fibrosis. In this study, an accelerated aging mouse model was established with D‐gal to determine the expression profile of TRPC3 and to identify the mechanism and role of TRPC3‐mediated mitochondrial dysfunction in renal disorders during aging.

## 2 RESULTS

### 2.1 Modulation of the Sirt1/AT1R axis improved renal disorders in accelerated aging mice

**FIGURE 1:**
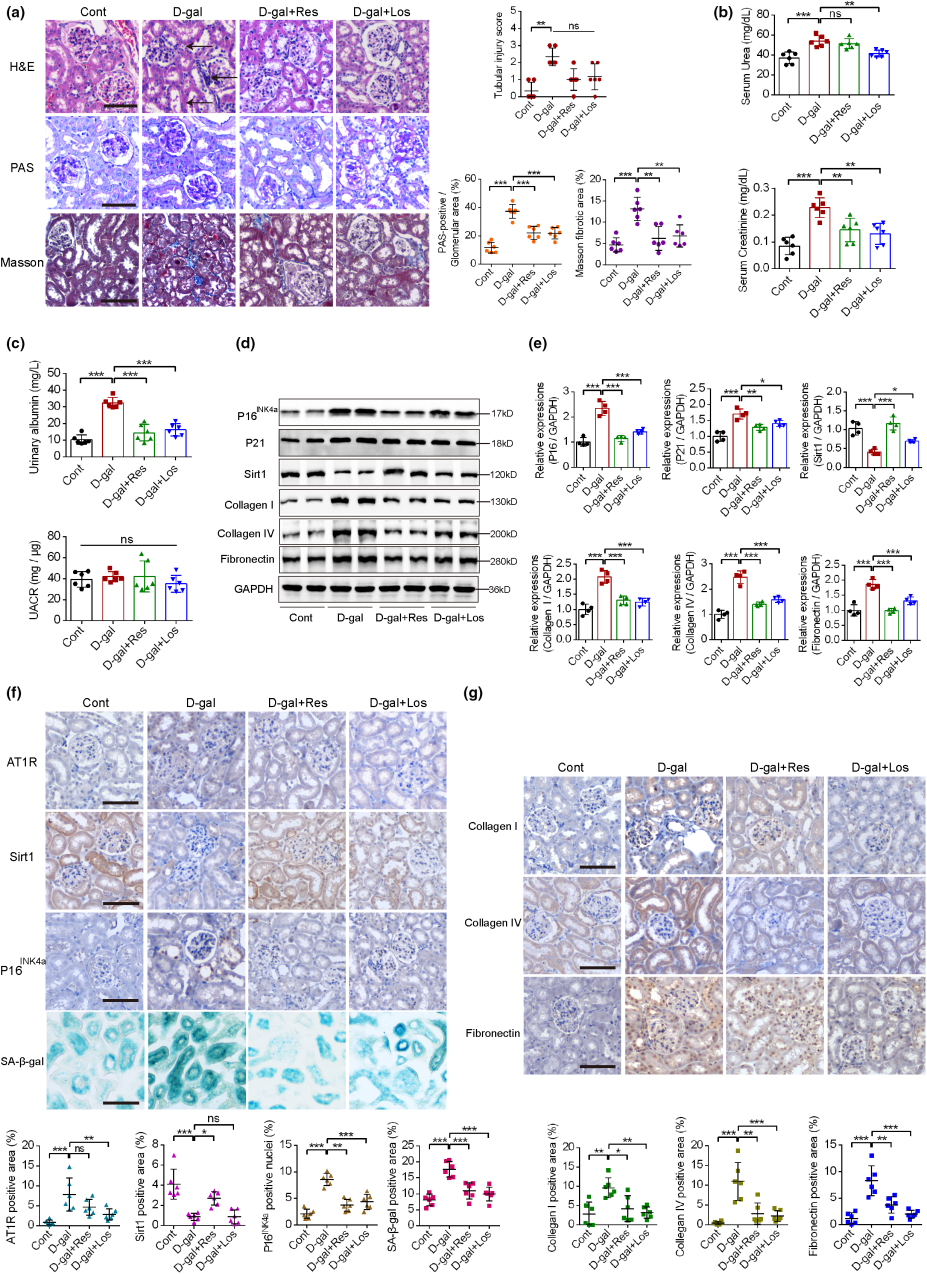
The effects of Sirt1 activation and AT1R inhibition on aging‐associated renal disorders. (a) Representative kidney sections of mice treated with control, D‐Galactose (D‐gal), D‐gal plus resveratrol and D‐gal plus losartan, stained with hematoxylin and eosin (H&E), periodic acid‐Schiff (PAS) and Masson dyes, with tubular injury score analysis, PAS‐positive area percentage and Masson fibrotic area percentage presented (*n* = 6). The arrows indicate tubular atrophy, glomerulosclerosis, and tubular cell death. (b) The effects of Sirt1 activation and AT1R inhibition on serum urea and creatinine levels of control and D‐gal treated mice (*n* = 6). (c) The effects of Sirt1 activation and AT1R inhibition on urinary albumin concentrations (mg/L) and urinary albumin/creatinine ratio (UACR, mg/μg) levels of control and D‐gal treated mice (*n* = 6). (d, e) The western blot analyses showed the expression profiles of senescence‐associated (P16^INK4a^, P21 and Sirt1), and fibrotic (Collagen I, Collagen IV, Fibronectin) markers in renal tissues, with glyceraldehyde‐3‐phosphate dehydrogenase (GAPDH) as a loading control in the above‐mentioned groups. Quantitative analysis of these bands was performed and calculated for statistical significance (*n* = 4). (f, g) Representative kidney sections of the above‐mentioned mice, with immunohistochemical staining of AT1R, Sirt1, P16^INK4a^, Collagen I, Collagen IV, Fibronectin. Also, representative kidney sections with senescence‐associated β‐galactosidase activity (SA‐β‐gal) staining were presented. Positive area percentage of these target proteins were analyzed by Image J (*n* = 6). *p < 0.05, **p < 0.01, ***p < 0.001.

We established an accelerated aging mouse model by subcutaneous D‐gal injection. As shown by hematoxylin and eosin (H&E) staining, control group kidneys displayed normal tissue structure, size, and glomerular shape and lacked inflammatory cell proliferation, atrophy, or sclerosis in the kidney tubules (Figure 1a). Injection of D‐gal resulted in glomerular sclerosis and cavity expansion and a decreased number of normal glomeruli. In addition, sclerosis, atrophy, and edema were observed in kidney tubules with inflammatory cell infiltration. However, these pathological changes were significantly attenuated upon sirtuin 1 (Sirt1) activation (resveratrol administration) or angiotensin II type 1 receptor (AT1R) inhibition (losartan administration). Histological assessment of periodic acid‐Schiff (PAS)‐stained renal sections revealed advanced, diffuse, and global sclerosis of glomeruli with glomerular matrix increase in the D‐gal‐injected group compared with the control group, while administration of resveratrol or losartan significantly improved these changes (Figure 1a). To clarify the degree of kidney fibrosis, renal sections were assessed with Masson's trichrome staining (Figure 1a). Significant differences in collagen accumulation in the kidney stroma and perivascular areas were observed between the control and D‐gal‐treated groups, while administration of resveratrol or losartan significantly decreased the collagen accumulation induced by D‐gal. Meanwhile, D‐gal intervention hampered renal functions, with increased serum urea and creatinine levels, while resveratrol or losartan supplementation improved these functions (Figure 1b). Urinary albumin concentrations and urinary albumin to urinary creatinine ratios (UACR) were measured. Resveratrol and losartan significantly decreased the upregulated urinary albumin concentrations induced by D‐gal, while UACRs remained unchanged among these groups (Figure 1c). To further quantify the renal senescence and kidney injuries, immunoblotting of key aging‐associated markers and renal fibrotic markers were conducted using renal tissues. D‐gal intervention upregulated the levels of Collagen I, Collagen IV, fibronectin, P16^INK4a^, and P21 while downregulated the levels of Sirt1. Intervention with resveratrol or losartan partially reversed these changes of key fibrotic and senescence‐associated markers (Figure 1d,e). Furthermore, the levels and distribution patterns of these proteins were detected using immunohistochemical staining of renal sections, with similar patterns observed (Figure 1f,g). To further support the observations in the D‐gal model, changes of key renal parameters were verified using both young mice (6 months of age) and naturally aged mice (22 months of age). Naturally aged mice exhibited increased glomerular sclerosis and cavity expansion with sclerosis, atrophy and edema in renal tubules with inflammatory cell infiltration (Figure S1a,b). Renal functions were impaired in aged mice, with increased urinary albumin concentrations, but the UACR remained unchanged (Figure S1c). Key parameters were measured using both immunoblots and immunohistochemistry, and the trends were similar to those in the D‐gal‐induced model (Figure S1d–g). Also, activation of Sirt1 and inhibition of AT1R signaling ameliorated the expression of senescence‐associated β‐galactosidase activity (SA‐β‐gal) in renal tubular epithelial cells compared with the D‐gal‐treated group (Figure 1f). These results indicate that modulation of Sirt1/AT1R signaling improved senescence‐associated renal disorders.

### 2.2 Modulation of the Sirt1/AT1R axis improved mitochondrial functions

**FIGURE 2:**
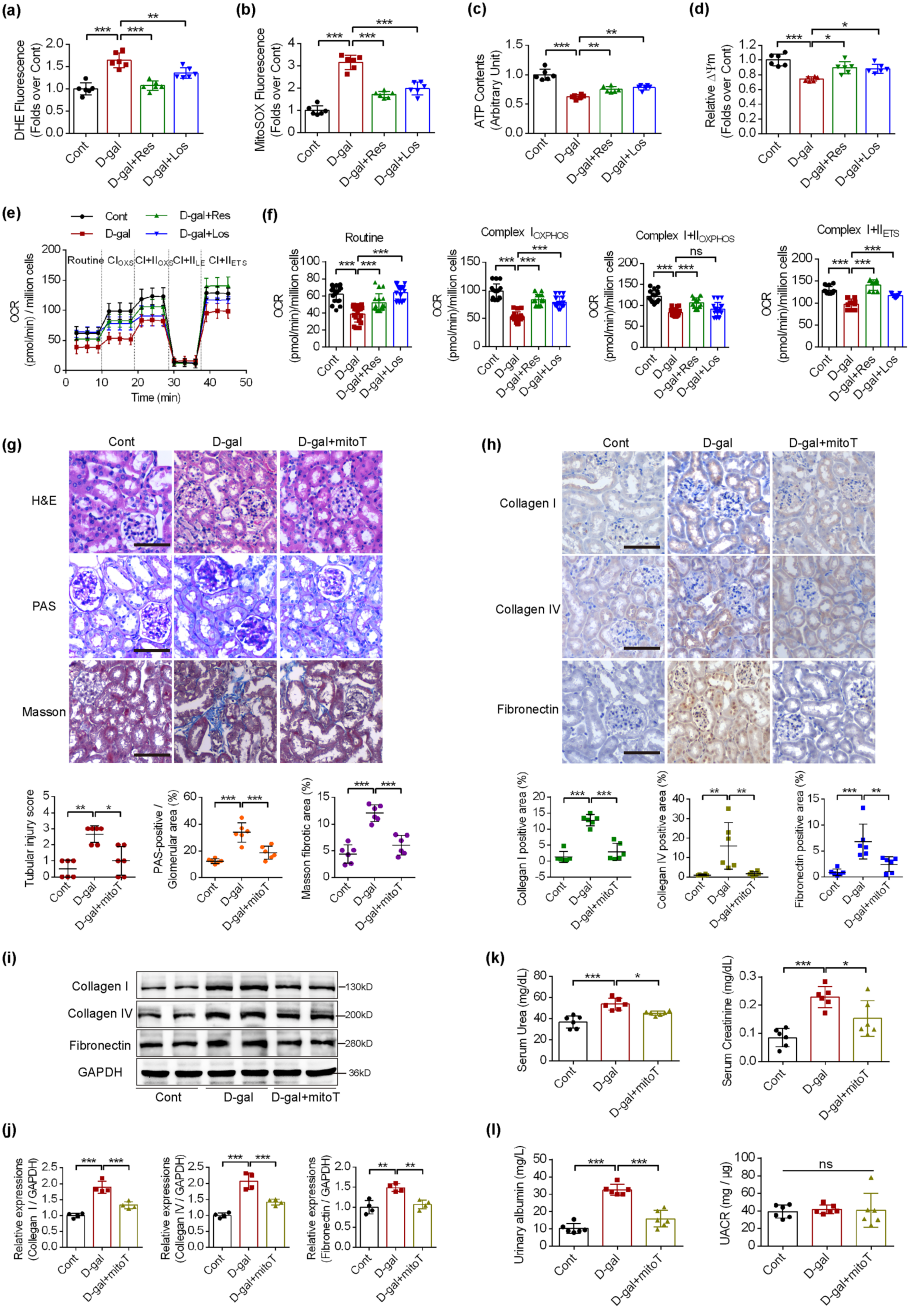
The associations between sirtuin 1/angiotensin II type 1 receptor (Sirt1/AT1R) signaling pathway and mitochondrial function. (a, b) The ROS levels in primary renal tubular epithelial cells (RTECs) from mice of control, D‐gal, D‐gal plus resveratrol and D‐gal plus losartan groups, using dihydroethidium (DHE) and mitoSOX as indicating dyes (*n* = 6). (c) The ATP contents of primary RTECs from the above‐mentioned groups (*n* = 6). (d) The mitochondrial membrane potential (MMP) levels of primary RTECs from the above‐mentioned groups, using JC‐1 as the indicating dye (*n* = 6). (e, f) The mitochondrial respiratory function of primary RTECs from the above‐mentioned groups. The Routine OCR levels, Complex I OXPHOS, Complex I plus II OXPHOS and ETS were calculated and analyzed in (f) (*n* = 9–18). (g) Representative kidney sections staining with hematoxylin and eosin (H&E), periodic acid‐Schiff (PAS) and Masson dyes from the above‐mentioned groups (*n* = 6). (h) Representative immunohistochemical kidney sections of mice treated with control, D‐gal, and D‐gal plus mitoTEMPO, stained with key fibrotic markers and quantified with Image J (*n* = 6). (i, j) The western blot analyses showed the expression levels of fibrotic markers (Collagen I, Collagen IV, Fibronectin), with GAPDH as a loading control in the above‐mentioned groups. Quantitative analysis of these bands was performed and calculated for statistical significance (*n* = 4). (k, l) The effects of mitoTEMPO intervention on renal functions, urinary albumin concentrations (mg/L), and urinary albumin/creatinine ratio (UACR, mg/μg) (*n* = 6). *p < 0.05, **p < 0.01, ***p < 0.001.

Sirt1 plays important roles in both regulation of mitochondrial function and aging; therefore, we measured the effects of Sirt1/AT1R on mitochondrial functions in RTECs. As shown in Figure S2, the purity of the RTEC population was more than 95% based on the morphology and cytokeratin 18 and E‐cadherin staining. Resveratrol and losartan intervention decreased the dihydroethidium (DHE) and mitoSOX fluorescence and improved the ATP content and mitochondrial membrane potential (MMP) in RTECs, which were affected by D‐gal intervention (Figure 2a–d). Moreover, high‐resolution respiratory evaluation was performed, revealing that resveratrol and losartan intervention improved routine oxygen consumption rate (OCR) values, Complex I‐dependent oxidative phosphorylation (CI_OXPHOS_), and Complex I plus II‐dependent oxidative phosphorylation (CI + II_OXPHOS_), which were suppressed by accelerated aging (Figure 2e,f). To directly demonstrate the role of mitochondrial ROS on aging‐associated renal disorders, a mitochondria‐specific ROS scavenger, mitoTEMPO, was use in vivo in the D‐gal model mice. MitoTEMPO is a new cell‐permeable mitochondria‐targeted antioxidant that eliminates mitochondrial superoxide and preserves MMP (Shetty et al., 2019). It consists of piperidine nitroxide TEMPOL conjugated with a positively charged triphenylphosphonium cation that facilitates 1000‐fold accumulation into the mitochondrial matrix (Dikalov, 2011). MitoTEMPO intervention reversed the glomerular sclerosis and tubular fibrosis induced by D‐gal, as shown by H&E, PAS, and Masson staining and by immunohistochemical staining of Collagen I, Collagen IV, and fibronectin (Figure 2g,h). Furthermore, immunoblotting of Collagen I, Collagen IV, and fibronectin showed the same trends as the immunohistochemical staining (Figure 2i,j). More importantly, mitoTEMPO intervention partially attenuated D‐gal‐induced impaired renal functions and increased urinary albumin concentrations (Figure 2k,l). These results indicate that Sirt1/AT1R modulation improved mitochondrial functions, which play important roles in the pathogenesis of aging‐associated renal disorders.

### 2.3 The expression patterns of TRPC channels in aging kidney upon resveratrol and losartan treatment

**FIGURE 3:**
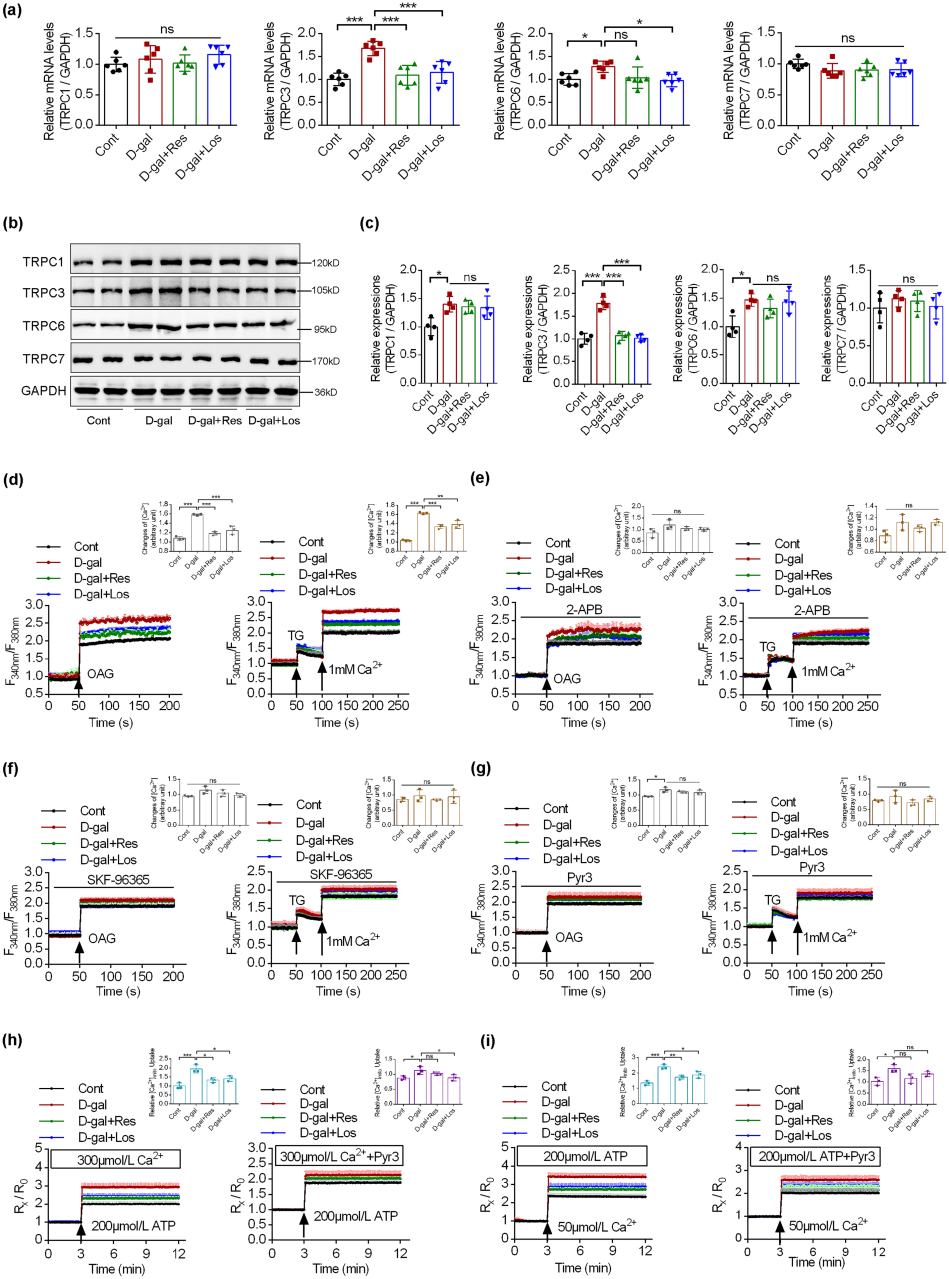
The expression profiles of TRPC channels and calcium influx patterns in aging renal tubular epithelial cells (RTECs). (a) QRT‐PCR evaluation of the mRNA levels of TRPC channels in renal tissues from mice of control, D‐gal, D‐gal plus resveratrol, and D‐gal plus losartan groups (*n* = 6). (b, c) The protein levels of TRPC channels using immunoblotting in renal tissues from the above‐mentioned mice (*n* = 4). (d) Cellular calcium influx was measured in primary RTECs using oleyl‐acetyl glycerol (OAG) and (thapsigargin) TG as agonists, respectively (*n* = 3). (e, f) The role of store‐operated calcium and TRPC channels on cellular calcium influx in primary RTECs by pre‐incubation of 2‐APB and SKF‐96365 as inhibitors, respectively (*n* = 3). (g) The role of transient receptor potential channel, subtype C, member 3 (TRPC3) on cellular calcium influx in primary RTECs by pre‐incubation of TRPC3 inhibitor Pyr3 (*n* = 3). (h, i) The role of TRPC3 on mitochondrial calcium entry in primary RTECs using complete RTECs (h) or digitonin‐treated permeabilized RTECs (i), in the absence or presence of Pyr3 (*n* = 3). *p < 0.05, **p < 0.01, ***p < 0.001.

TRPC channels function as a link between intracellular calcium and mitochondrial functions (Nan et al., 2021); therefore, the renal expression patterns of TRPC channels were analyzed in the D‐gal model mice. The mRNA levels of TRPC channels in renal tissues were measured using quantitative reverse‐transcription polymerase chain reaction (qRT‐PCR). The mRNA levels of TRPC3 and TRPC6 were upregulated upon D‐gal treatment, while those of TRPC1 and TRPC7 did not change. Upon D‐gal intervention, only TRPC3 mRNA levels were significantly decreased upon resveratrol or losartan treatment (Figure 3a). We also detected TRPC protein levels using immunoblotting. Similar trends were exhibited for protein levels, showing that TRPC3 and TRPC6 were upregulated upon D‐gal intervention, while resveratrol or losartan treatment partially reversed TRPC3 upregulation (Figure 3b,c). TRPC6 protein levels also manifested a decreased trend upon resveratrol or losartan treatment, but this change was not statistically significant. These results indicate that TRPC3 might be a key factor linking calcium homeostasis and mitochondrial function in aging.

### 2.4 TRPC3 plays important roles in cellular and mitochondrial calcium homeostasis in aging kidney

We next investigated the key players in modulating calcium homeostasis in aging RTECs. Using oleyl‐acetyl glycerol (OAG) to activate calcium influx through TRPC channels, we showed that D‐gal intervention increased the calcium influx induced by OAG in primary RTECs, while resveratrol or losartan treatment partially reversed these trends (Figure 3d). Meanwhile, using thapsigargin (TG) to induce store‐operated calcium entry (SOCE), we demonstrated that resveratrol or losartan treatment partially reversed the increased SOCE, induced by D‐gal in primary RTECs (Figure 3d). 2‐APB or SKF‐96365 were used to inhibit SOCE and TRPC channels‐induced calcium influx and showed no significant difference among the four groups (Figure 3e,f). More specifically, a TRPC3 inhibitor, Pyr3, was added and inhibited TG‐induced calcium influx and partially inhibited OAG‐induced calcium influx (Figure 3g). TRPC3 can function as both a store‐operated calcium channel and a receptor‐operated channel (Liu et al., 2007); therefore, these results indicate that TRPC channels, especially TRPC3, play important roles in dysregulated calcium homeostasis upon D‐gal intervention. To better demonstrate the role of mitochondrial TRPC3 in calcium regulation, ATP was added to induce mitochondrial calcium influx. Figure 3h (untreated cells) and Figure 3i (digitonin‐treated permeabilized cells) show that D‐gal intervention also induced dramatic increases in mitochondrial calcium influx, while Pyr3 treatment greatly suppressed those calcium influxes in primary RTECs. Together, these results demonstrate that TRPC3 functions during dysregulated cellular and mitochondrial calcium influx upon D‐gal intervention.

### 2.5 Increased TRPC3 expression hampers mitochondrial functions in aging kidney

**FIGURE 4:**
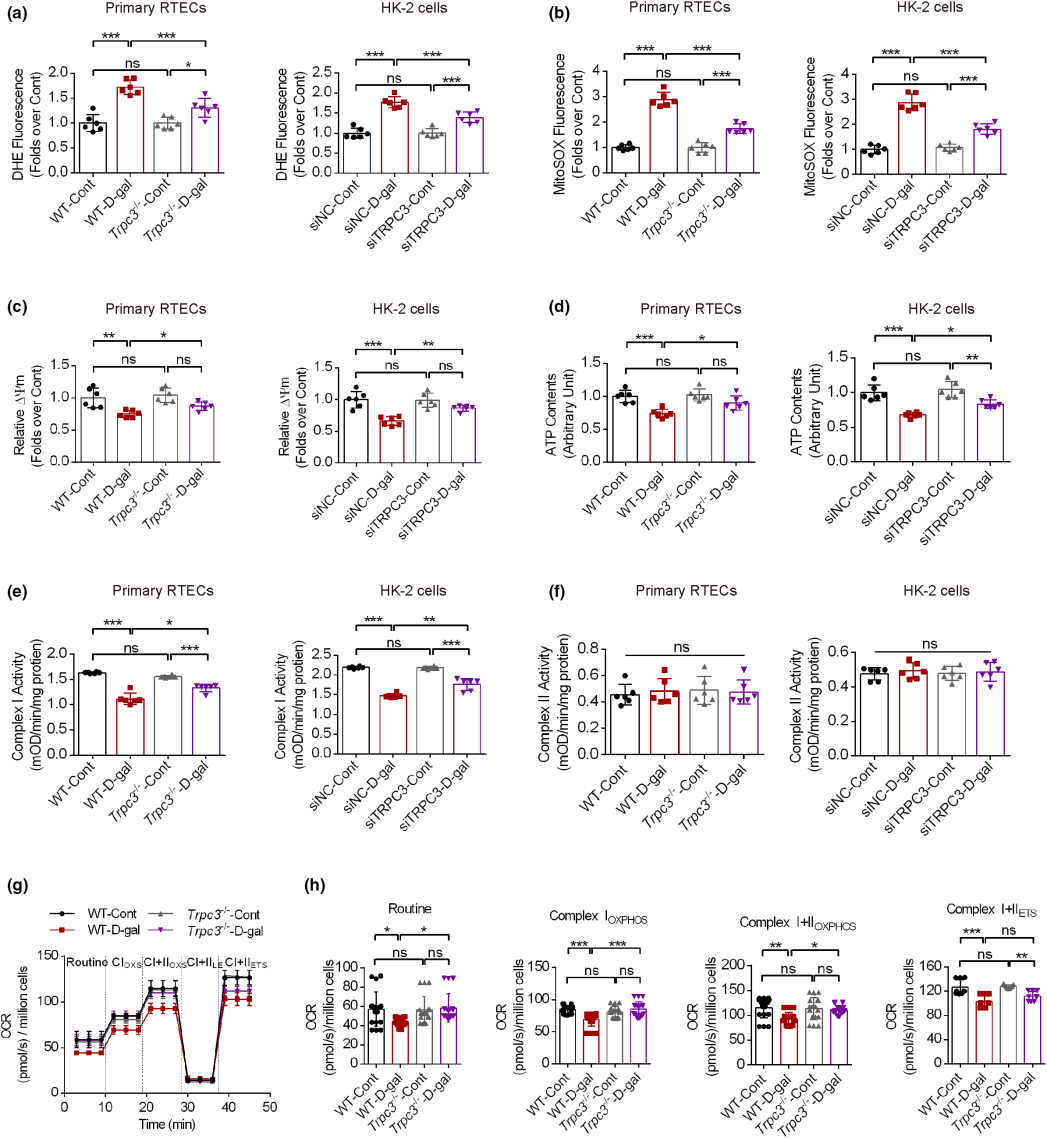
The effects of transient receptor potential channel, subtype C, member 3 (TRPC3) on mitochondrial function in aging renal tubular epithelial cells (RTECs). (a, b) Cellular and mitochondrial ROS levels were detected using dihydroethidium (DHE) and mitoSOX as fluorescence dyes in primary RTECs and HK‐2 cells (treated with siNC/siTRPC3), respectively (*n* = 6). (c, d) The mitochondrial membrane potential (MMP) levels and ATP contents were measured in the above‐mentioned RTECs (*n* = 6). (e, f) The enzyme activities of Complex I and Complex II in mitochondrial respiratory chain were measured in both primary RTECs and HK‐2 cells (*n* = 6). (g, h) Mitochondrial respiratory functions were measured in primary RTECs (*n* = 9–18). *p < 0.05, **p < 0.01, ***p < 0.001.

To better validate the role of TRPC3 in regulating mitochondrial functions, both primary RTECs from wild‐type and *Trpc3* ^*−/−*^ mice and HK‐2 cells were used with D‐gal intervention. D‐gal treatment upregulated both DHE and mitoSOX fluorescence intensity in primary RTECs and HK‐2 cells. *Trpc3* deficiency significantly decreased the fluorescence intensity of both DHE and mitoSOX upon D‐gal intervention, while no significant effects were observed between wild‐type/siNC and *Trpc3* ^*−/−*^/siTRPC3 groups in untreated RTECs (Figure 4a,b). Moreover, *Trpc3* deficiency partially reversed the decreased MMP levels and ATP production upon D‐gal treatment, with no significant difference in MMP and ATP values between wild‐type/siNC and *Trpc3* ^*−/−*^/siTRPC3 groups in untreated RTECs (Figure 4c,d). The enzyme activities of Complex I and II from the mitochondrial respiratory chain were then measured. D‐gal intervention inhibited the enzyme activity of Complex I in both RTECs and HK‐2 cells, while *Trpc3* knockout (KO) or knockdown partially recovered the enzyme activity (Figure 4e,f). No significant difference between untreated wild‐type/siNC and *Trpc3* ^*−/−*^/siTRPC3 groups was observed regarding Complex I enzyme activity. No significant difference in Complex II enzyme activity was observed these groups. The mitochondrial respiratory functions were then assessed. The routine OCR, CI_OXPHOS_, CI + II_OXPHOS_, and Complex I plus II‐supported noncoupled respiration (CI + II_ETS_) values in primary RTECs were reduced upon D‐gal treatment, with *Trpc3* KO partially reversing these parameters (Figure 4g,h). Intestinally, *Trpc3* deficiency greatly ameliorates the above‐mentioned changes induced by D‐gal intervention by comparing the *Trpc3* deficient groups with or without D‐gal intervention. These results indicate that TRPC3 upregulation‐mediated calcium overload promoted mitochondrial dysfunction in aging RTECs.

### 2.6 Dysregulated AT1R/PKA/CREB pathway is responsible for enhanced TRPC3 transcription in HK‐2 cells

**FIGURE 5:**
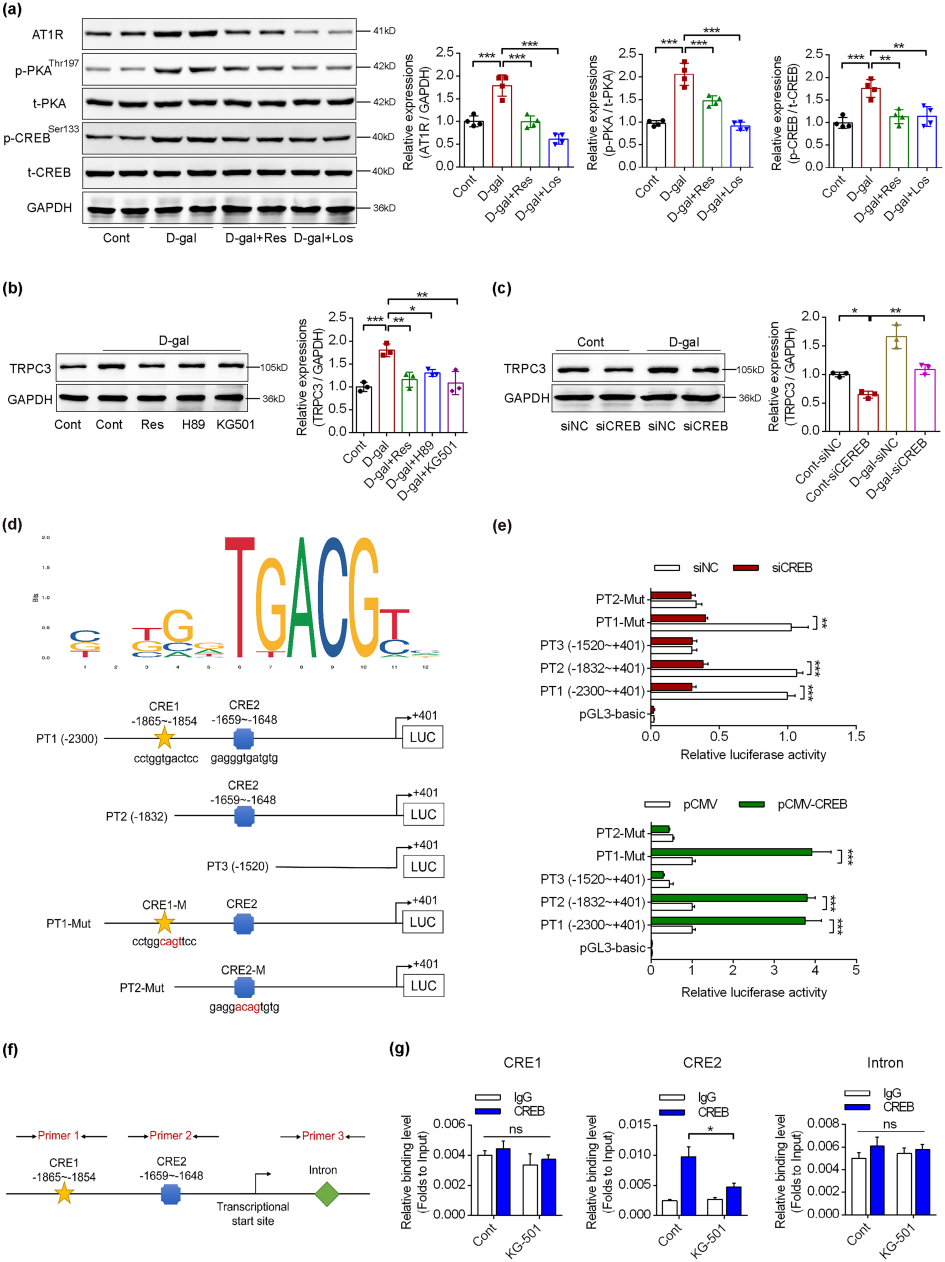
The regulatory mechanism of enhanced transient receptor potential channel, subtype C, member 3 (TRPC3) transcription in renal tubular epithelial cells (RTECs). (a) Immunoblotting detection of the key proteins in the (sirtuin 1/angiotensin II type 1 receptor) Sirt1/AT1R signaling pathway of the renal tissues from the above‐mentioned groups, using GAPDH as a loading control (*n* = 4). (b, c) The effects of Sirt1/AT1R signaling pathway on TRPC3 levels by combined usage of inhibitors and siRNAs transfection (*n* = 3). (d) The possible CRE binding sequence and location in the TRPC3 promoter region by JASPAR database, with the schematic diagram of different recombinant vectors used for dual‐luciferase reporter assay. (e) Dual‐luciferase reporter assay was performed by co‐transfection of recombinant vectors, siNC/siCREB or pCMV/pCMV‐cAMP‐response element binding protein (CREB), and pRL‐TK plasmids in HK‐2 cells (*n* = 3). (f, g) Chromatin immunoprecipitation (ChIP)‐PCR assay to demonstrate the direct binding site in the TRPC3 promoter region (*n* = 3). *p < 0.05, **p < 0.01, ***p < 0.001.

To investigate the detailed mechanisms of enhanced TRPC3 expression, we detected the expression profiles of key signaling pathway components upstream of TRPC3. We observed enhanced levels of AT1R, and phosphorylation of protein kinase A (PKA) and cAMP‐response element binding protein (CREB) upon D‐gal intervention, while resveratrol or losartan partially attenuated these changes (Figure 5a). We then examined the effects of inhibitors on the expression of TRPC3. The Sirt1 activator, resveratrol, PKA inhibitor, H89, and CREB inhibitor, KG501, partially blunted the upregulation of TRPC3 induced by D‐gal (Figure 5b). CREB knockdown also inhibited the upregulation of TRPC3 by D‐gal treatment in HK‐2 cells, compared with siNC (Figure 5c). Using the JASPAR database for promoter prediction, we identified a predicted binding sequence of CRE with two possible CRE binding sites, located at −1865 to −1854 bp and −1659 to −1648 bp (Figure 5d). Dual‐luciferase reporter assays showed that CREB knockdown inhibited the luciferase activity of the recombinant reporters, PT1, PT2 and PT1‐Mut, compared with siNC, while no significant changes were observed after transfection of pGL3‐basic, PT3 or PT2‐Mut, indicating that CRE2 is the potential binding site. Co‐transfection of pCMV‐CREB showed similar trends, further demonstrating that CRE2 is the binding site of CREB (Figure 5e). Furthermore, chromatin immunoprecipitation (ChIP)‐PCR assay revealed that CREB can directly bind to the TRPC3 promoter region (−1659 to −1648 bp) (Figure 5f,g). These findings indicate that activation of the AT1R/PKA pathway promoted the binding of CREB to the TRPC3 promoter to activate transcription.

### 2.7 *Trpc3* KO further ameliorates aging‐associated renal disorders

**FIGURE 6:**
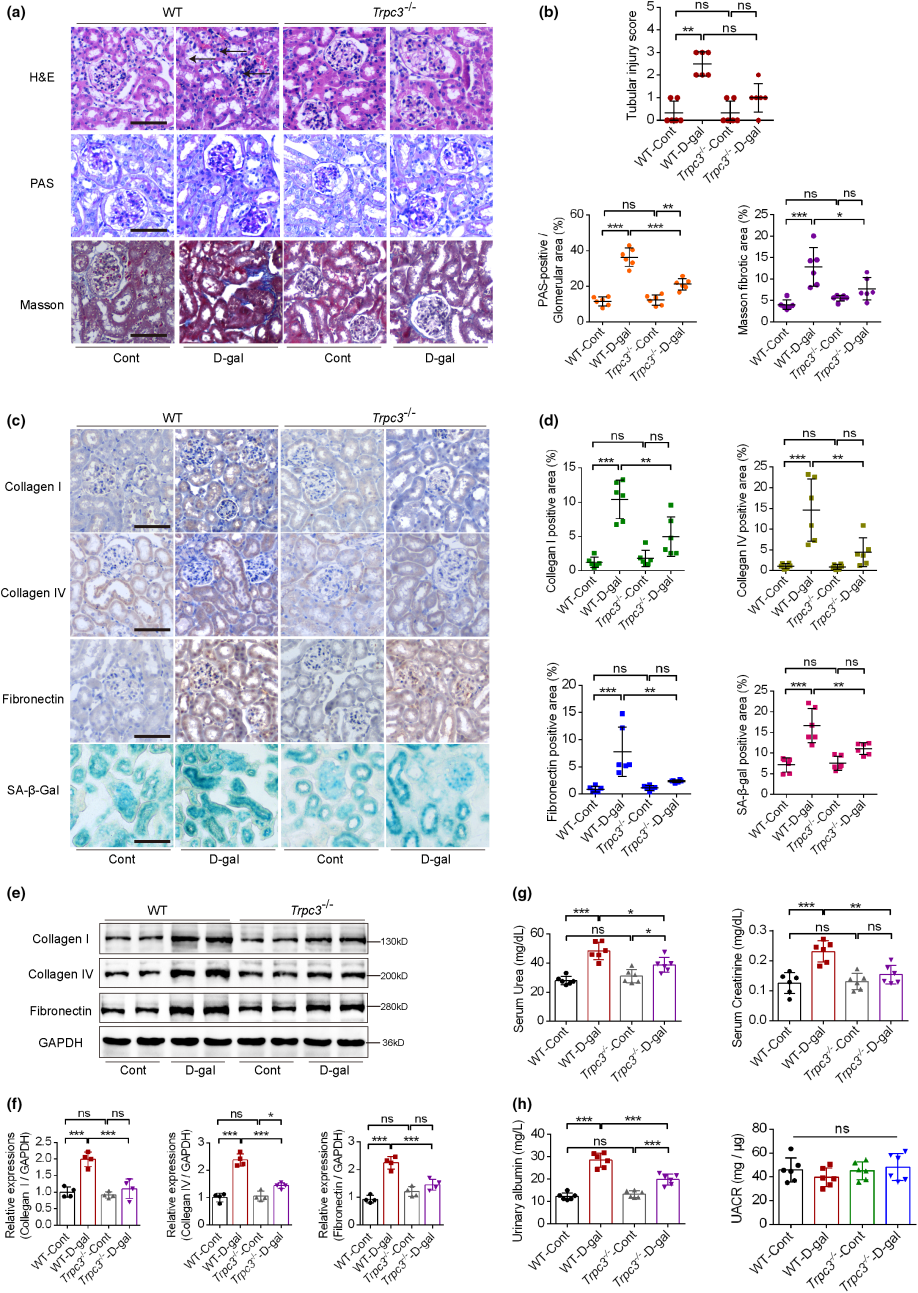
The effects of *Trpc3* deficiency on aging‐associated renal disorders. (a, b) Representative graphs of renal sections stained with hematoxylin and eosin (H&E), periodic acid‐Schiff (PAS) and Masson dyes in both wild‐type and *Trpc3* KO mice treated with D‐gal (*n* = 6). The arrows indicate tubular atrophy, renal tubular epithelial cells (RTECs) loss and glomerulosclerosis. (c, d) Representative renal immunohistochemical sections of the above‐mentioned mice, stained with key fibrotic markers and quantified with Image J (*n* = 6). (e, f) Immunoblotting evaluation of fibrotic marker levels (Collagen I, Collagen IV, Fibronectin), with GAPDH as a loading control in the above‐mentioned mice. Quantitative analysis of these bands was performed and calculated for statistical significance (*n* = 4). (g, h) The effects of *Trpc3* deficiency on renal functions, urinary albumin (mg/L), and urinary albumin/creatinine ratio (UACR, mg/μg) levels (*n* = 6). *p < 0.05, **p < 0.01, ***p < 0.001.

To further validate the role of TRPC3 in modulating aging‐associated renal disorders, both wild‐type and *Trpc3* ^*−/−*^ mice were treated with D‐gal. *Trpc3* KO partially ameliorated the glomerular sclerosis, tubular atrophy, and renal interstitial fibrosis induced by D‐gal intervention (Figure 6a,b). Moreover, increased levels of key fibrotic markers (Collagen I, Collagen IV and fibronectin) were determined to be partially attenuated by *Trpc3* KO using both immunohistochemical staining and western blotting. *Trpc3* KO also partially decreased the size of the SA‐β‐gal positive area induced by D‐gal intervention (Figure 6c–f). Furthermore, *Trpc3* KO partially attenuated D‐gal‐induced impaired renal functions and increased urinary albumin concentrations (Figure 6g,h). These findings provide further evidence of TRPC3 modulating aging‐associated renal disorders.

## 3 DISCUSSION

Investigating the detailed mechanisms of aging‐associated mitochondrial dysfunction and renal disorders is vital to understand the pathogenesis senescence‐related CKD. In this study, we demonstrated that modulation of the Sirt1/AT1R axis improved aging‐associated mitochondrial function and renal disorders, effects that are associated with TRPC3 upregulation. TRPC3 upregulation induced dysregulated cellular/mitochondrial calcium homeostasis and mitochondrial dysfunction, which promoted oxidative stress and pathogenesis of renal fibrosis. *Trpc3* KO partially attenuated D‐gal‐induced renal disorders by improving mitochondrial function. Our study reveals a new mechanism of dysregulated redox status and impaired mitochondrial function, which promotes cell senescence in the aging kidney. TRPC3 may also be an important target to improve mitochondrial function and aging‐associated renal injury.

There are various types of in vivo accelerated aging model, namely radiation‐induced, jet lag‐induced, the Klotho mouse, thymus‐removed, and D‐gal‐induced models (Azman & Zakaria, 2019). Among them, the D‐gal‐induced model is the most preferred and widely used in the study of aging. Chronically administered D‐gal is converted into aldose and hydroperoxide by galactose oxidase, resulting in the generation of ROS, inflammation, mitochondrial dysfunction, and apoptosis (Ullah et al., 2015). The D‐gal‐induced aging model has been used to mimic natural aging and to investigate various aging‐associated dysfunctions, such as systemic changes, brain aging, liver aging, and kidney aging (Hong et al., 2023; Miao et al., 2019; Sumbalová et al., 2022). In the naturing process of aging, impaired redox homeostasis is considered to play a dominant role. D‐gal intervention mimics this aging process by inducing oxidative stress in kidney tissues through the elevation of renal malondialdehyde, nitric oxide, protein carbonyl and ROS levels while diminishing superoxide dismutase, catalase and glutathione levels (E et al., 2023). Oxidative stress may further activate a variety of inflammatory signaling pathways and lead to the expression of inflammatory cytokines (Azman & Zakaria, 2019). Moreover, D‐gal administration significantly increases the levels of serum urea and creatinine, and can lead to histological changes in the kidney (Gao et al., 2022). Azman & Zakaria also concluded that the extent of damage is different between D‐gal‐induced and natural aging but that this damage is not easy to measure (Azman & Zakaria, 2019). Therefore, the D‐gal‐induced aging model is a relatively good model to mimic natural aging and to investigate its mechanisms.

Human aging is associated with both structural and functional changes in various organ systems, including the kidney. During the aging process, the kidney exhibits progressive functional decline with histological alterations (Fang et al., 2020). Renal tubules account for more than 90% of the total renal mass and are mostly affected by kidney ageing; therefore, RTECs are challenged by both intrinsic (replicative) senescence and extrinsic (stress‐induced) senescence (Sturmlechner et al., 2017). Recent studies indicate that senescence of RTECs accelerates the progression of renal fibrosis (Wang et al., 2021). Mechanistically, senescent RTECs may secrete senescence‐associated secretory phenotype (pro‐fibrotic and pro‐inflammatory) factors, further promoting the senescence of renal cells, and accelerating renal aging (Docherty et al., 2019). Zhang et al. concluded that oxidative stress and telomere attrition with DNA damage are the major causes of RTEC senescence, with oxidative stress acting as the initiation step (Zhang et al., 2023). Moreover, Wnt/β‐catenin/renin‐angiotensin system (RAS), nuclear factor erythroid 2‐related factor 2 (Nrf2)/ARE and signal transducer and activator of transcription‐3 (STAT‐3)/NF‐kB pathways are involved in the pathogenesis of renal disorders during aging (Zhang et al., 2023). The detailed mechanism underlying these pathways have been studied. For instance, Miao et al. revealed that the Wnt/RAS axis functions in age‐related renal fibrosis through the deterioration of mitochondrial function and redox status (Miao et al., 2019). In this study, we revealed the downstream mechanisms of Sirt1/AT1R signaling involvement in redox status and pathogenesis of aging‐associated renal disorders.

Mitochondrial dysfunction and ROS production are central to RTEC senescence (Zhang et al., 2023). Mitochondria are fundamental to energy metabolism and redox homeostasis; therefore, their dysfunction is a crucial underlying factor in aging. Aging impairs mitochondrial oxidative phosphorylation in heart tissue, with an approximately 50% decrease in function, especially of Complex I (Lesnefsky et al., 2016). Moreover, impaired mitochondria during aging are major producers of excessive ROS, and prime targets for excessive ROS‐induced damage, leading to deterioration of oxidative phosphorylation and redox balance. Changes to mitochondria during senescence include changes in mitochondrial morphology/function, OXPHOS function, and calcium homeostasis. For instance, dysregulation of the Wnt/β‐catenin/RAS pathway promotes mitochondrial dysfunction and renal fibrosis during aging (Miao et al., 2019). The mitochondrial protein, Fus1/Tusc2, also functions in age‐related pathologies by modulating energy homeostasis (Uzhachenko et al., 2017). Therefore, mitochondrial metabolic reprogramming has been proposed to be an important therapeutic strategy for aging and age‐related diseases (Chung et al., 2018; Lee et al., 2021). In this study, mitochondrial functions in D‐gal‐treated RTECs were systematically assessed in aged renal tissues, and showed increased ROS production, impaired ATP production and OXPHOS, and decreased MMP levels. More importantly, we showed that D‐gal‐induced mitochondrial dysfunction and aging‐associated renal disorders can be partially attenuated by modulation of Sirt1/AT1R signaling or by knockdown of TRPC3.

Among the various alterations to mitochondrial functions during aging, changes in Ca^2+^ homeostasis appear to initiate these alterations (Müller et al., 2018). Mitochondria are important calcium stores in the cell and are affected by the calcium status of the whole cell (Bou‐Teen et al., 2021). Generally, dysregulated calcium homeostasis leads to mitochondrial calcium overload through multiple calcium channels located in the mitochondrial membrane, such as mitochondrial calcium uniporter. A mutual interplay between ROS and Ca^2+^ in aging and age‐related diseases has been suggested (Madreiter‐Sokolowski et al., 2020). Therefore, dysregulated calcium homeostasis has been shown to function in aging‐associated disorders. Previously, increased cytosolic Ca^2+^ levels were observed in aging MSCs (Ahamad et al., 2021), and were correlated with age‐related diseases (Madreiter‐Sokolowski et al., 2020). Moreover, targeting mitochondrial calcium homeostasis is a therapeutic strategy to improve aging‐associated diseases (Gao et al., 2020; Lee et al., 2021). We previously demonstrate that TRPC3 functions to regulate mitochondrial calcium uptake and mitochondrial function in hypertensive vasculature (Wang et al., 2017). TRPC3 is different from other calcium channels because it is located in both the plasma and mitochondrial membranes (Feng et al., 2013). Therefore, TRPC3 upregulation may directly lead to mitochondrial calcium overload, with the mitochondrial calcium imbalance resulting in mitochondrial and redox dysfunctions. However, the role of TRPC3 has not been investigated in aging‐associated renal changes. Erac et al. have reported increased expressions of TRPC1 and TRPC6 in aging rat aorta (Erac et al., 2010). He et al. reported that upregulation of TRPC3 induced atrial fibrosis in both spontaneously hypertensive and aging rats (He et al., 2019). Here, we investigated the expression profiles of TRPC channels and showed that TRPC3 exhibited the largest increase in both mRNA and protein levels, while TRPC1 and TRPC6 showed a mild increase in protein levels. Interestingly, the mRNA level of TRPC1 did not change significantly, indicating that TRPC1 mRNA translation may be affected during aging, which is a promising avenue to pursue in the study of renal diseases (Kasinath et al., 2009). Furthermore, we showed that upregulation of TRPC3 promotes both cytosolic and mitochondrial calcium overload, mitochondrial dysfunction, and redox imbalance, thereby promoting aging‐associated renal fibrosis. Inhibition of TRPC3 by Pyr3 or *Trpc3* knockdown/KO partially attenuated cytosolic/mitochondrial calcium influx, ROS production, and mitochondrial respiratory dysfunction. Moreover, *Trpc3* KO partially ameliorated D‐gal‐induced CKD, evidenced by improved renal function with reduced pathological changes and renal fibrosis. Furthermore, the mechanism of TRPC3 upregulation was revealed in RTECs as dysregulated AT1R/PKA/CREB signaling. TRPC3 functions as a link between calcium entry, mitochondrial metabolism and ROS in aging kidney and is a promising therapeutic target for aging‐associated renal disorders.

TRPC3 and TRPC6 are both involved in several physiological processes and the pathogenesis of diseases. TRPC3 and TRPC6 are store‐operated calcium channels and form functional heteromultimers in the plasma membrane (Dryer et al., 2019). The roles of TRPC3 and TRPC6 have also been investigated in renal fibrosis (Wu et al., 2017) and CKD (Dryer et al., 2019). More specifically, TRPC3 and TRPC6 may play different roles in certain circumstances. For instance, increased TRPC3 expression in vascular smooth muscle cells leads to changes in TRPC3/C6 heteromultimeric assembly and promotes depolarization of hypertensive vascular smooth muscle cells (Álvarez‐Miguel et al., 2017). TRPC6 can counteract the TRPC3‐Nox2 protein complex, thereby leading to decreased levels of cytokines and attenuation of hyperglycemia‐induced heart failure in mice (Oda et al., 2017). Furthermore, mutual interaction also exists between the expression patterns of TRPC3 and TRPC6. For instance, Dietrich et al. reported that the expression of TRPC3 was upregulated upon TRPC6 KO in smooth muscle cells (Dietrich et al., 2005). Therefore, the detailed regulatory mechanisms between TRPC3 and TRPC6 need further investigation, especially in kidney diseases.

In summary, dysregulation of the Sirt1/AT1R pathway, which induced PKA/CREB activation to promote TRPC3 transcription, was observed in RTECs during aging. Enhanced TRPC3 expression induced cellular calcium overload, mitochondrial dysfunction, and ROS production, thereby promoting the pathogenesis of renal disorder during aging. TRPC3 is a promising therapeutic target for aging‐associated renal disorders.

## AUTHOR CONTRIBUTIONS

Y‐Z.C., X‐M.C., G‐Y.C., B.W. and W‐P.Y. conceived and designed the study. B.W., W‐P.Y., Y‐Z.C., W‐G.Z., M.Z., Y.N., X‐Y.J. and J.Z. performed the experiments and analyzed the data. D.S., H.L., Z‐H.Z., Q.L., X‐W.C. and J‐X.N. provided key reagents and experimental protocols. B.W. and W‐P.Y. wrote the draft of the manuscript. G‐Y.C. contributed greatly to the manuscript revision. Y‐Z.C., X‐M.C. and G‐Y.C. reviewed the manuscript and supervised the study. All the authors discussed the results and approved the submission of final version of the manuscript.

## CONFLICT OF INTEREST STATEMENT

The authors declared no conflict of interest.

## Supporting information


Data S1:

## Data Availability

The data that support the findings of this study are available from the corresponding author upon reasonable request.

## References

[acel14130-bib-0001] Ahamad, N. , Sun, Y. , & Singh, B. B. (2021). Increasing cytosolic Ca(2+) levels restore cell proliferation and stem cell potency in aged MSCs. Stem Cell Research, 56, 102560. 10.1016/j.scr.2021.102560 34624617 PMC8596392

[acel14130-bib-0002] Álvarez‐Miguel, I. , Cidad, P. , Pérez‐García, M. T. , & López‐López, J. R. (2017). Differences in TRPC3 and TRPC6 channels assembly in mesenteric vascular smooth muscle cells in essential hypertension. The Journal of Physiology, 595(5), 1497–1513. 10.1113/jp273327 27861908 PMC5330869

[acel14130-bib-0003] Azman, K. F. , & Zakaria, R. (2019). D‐galactose‐induced accelerated aging model: An overview. Biogerontology, 20(6), 763–782. 10.1007/s10522-019-09837-y 31538262

[acel14130-bib-0004] Bou‐Teen, D. , Kaludercic, N. , Weissman, D. , Turan, B. , Maack, C. , Di Lisa, F. , & Ruiz‐Meana, M. (2021). Mitochondrial ROS and mitochondria‐targeted antioxidants in the aged heart. Free Radical Biology & Medicine, 167, 109–124. 10.1016/j.freeradbiomed.2021.02.043 33716106

[acel14130-bib-0005] Chung, K. W. , Lee, E. K. , Lee, M. K. , Oh, G. T. , Yu, B. P. , & Chung, H. Y. (2018). Impairment of PPARα and the fatty acid oxidation pathway aggravates renal fibrosis during aging. Journal of the American Society of Nephrology, 29(4), 1223–1237. 10.1681/asn.2017070802 29440279 PMC5875952

[acel14130-bib-0006] Dietrich, A. , Mederos, Y. S. M. , Gollasch, M. , Gross, V. , Storch, U. , Dubrovska, G. , Obst, M. , Yildirim, E. , Salanova, B. , Kalwa, H. , Essin, K. , Pinkenburg, O. , Luft, F. C. , Gudermann, T. , & Birnbaumer, L. (2005). Increased vascular smooth muscle contractility in TRPC6−/− mice. Molecular and Cellular Biology, 25(16), 6980–6989. 10.1128/mcb.25.16.6980-6989.2005 16055711 PMC1190236

[acel14130-bib-0007] Dikalov, S. (2011). Cross talk between mitochondria and NADPH oxidases. Free Radical Biology & Medicine, 51(7), 1289–1301. 10.1016/j.freeradbiomed.2011.06.033 21777669 PMC3163726

[acel14130-bib-0008] Docherty, M. H. , O'Sullivan, E. D. , Bonventre, J. V. , & Ferenbach, D. A. (2019). Cellular senescence in the kidney. Journal of the American Society of Nephrology, 30(5), 726–736. 10.1681/asn.2018121251 31000567 PMC6493983

[acel14130-bib-0009] Dryer, S. E. , Roshanravan, H. , & Kim, E. Y. (2019). TRPC channels: Regulation, dysregulation and contributions to chronic kidney disease. Biochimica et Biophysica Acta‐Molecular Basis of Disease, 1865(6), 1041–1066. 10.1016/j.bbadis.2019.04.001 30953689

[acel14130-bib-0040] E, Y. , Lin, Y. , Yan, G. , Yang, J. , Jiao, L. , Wu, R. , Yan, Q. , Chen, Y. , Chen, Y. , Yan, X. , & Li, H. (2023). Exogenous H(2)S alleviates senescence of glomerular mesangial cells through up‐regulating mitophagy by activation of AMPK‐ULK1‐PINK1‐parkin pathway in mice. Biochimica et Biophysica Acta, Molecular Cell Research, 1870(8), 119568. 10.1016/j.bbamcr.2023.119568 37597773

[acel14130-bib-0010] Earley, S. , & Brayden, J. E. (2015). Transient receptor potential channels in the vasculature. Physiological Reviews, 95(2), 645–690. 10.1152/physrev.00026.2014 25834234 PMC4551213

[acel14130-bib-0011] Erac, Y. , Selli, C. , Kosova, B. , Akcali, K. C. , & Tosun, M. (2010). Expression levels of TRPC1 and TRPC6 ion channels are reciprocally altered in aging rat aorta: Implications for age‐related vasospastic disorders. Age (Dordrecht, Netherlands), 32(2), 223–230. 10.1007/s11357-009-9126-z 20431989 PMC2861749

[acel14130-bib-0012] Fang, Y. , Gong, A. Y. , Haller, S. T. , Dworkin, L. D. , Liu, Z. , & Gong, R. (2020). The ageing kidney: Molecular mechanisms and clinical implications. Ageing Research Reviews, 63, 101151. 10.1016/j.arr.2020.101151 32835891 PMC7595250

[acel14130-bib-0013] Feng, S. , Li, H. , Tai, Y. , Huang, J. , Su, Y. , Abramowitz, J. , Zhu, M. X. , Birnbaumer, L. , & Wang, Y. (2013). Canonical transient receptor potential 3 channels regulate mitochondrial calcium uptake. Proceedings of the National Academy of Sciences of the United States of America, 110(27), 11011–11016. 10.1073/pnas.1309531110 23776229 PMC3704010

[acel14130-bib-0014] Gao, P. , Jiang, Y. , Wu, H. , Sun, F. , Li, Y. , He, H. , Wang, B. , Lu, Z. , Hu, Y. , Wei, X. , Cui, Y. , He, C. , Wang, L. , Zheng, H. , Yang, G. , Liu, D. , Yan, Z. , & Zhu, Z. (2020). Inhibition of mitochondrial calcium overload by SIRT3 prevents obesity‐or age‐related whitening of Brown adipose tissue. Diabetes, 69(2), 165–180. 10.2337/db19-0526 31712319

[acel14130-bib-0015] Gao, Q. , Chen, F. , Zhang, L. , Wei, A. , Wang, Y. , Wu, Z. , & Cao, W. (2022). Inhibition of DNA methyltransferase aberrations reinstates antioxidant aging suppressors and ameliorates renal aging. Aging Cell, 21(1), e13526. 10.1111/acel.13526 34874096 PMC8761007

[acel14130-bib-0016] He, R. , Zhang, J. , Luo, D. , Yu, Y. , Chen, T. , Yang, Y. , Yu, F. , & Li, M. (2019). Upregulation of transient receptor potential canonical type 3 channel via AT1R/TGF‐β1/Smad2/3 induces atrial fibrosis in aging and spontaneously hypertensive rats. Oxidative Medicine and Cellular Longevity, 2019, 4025496. 10.1155/2019/4025496 31871548 PMC6906806

[acel14130-bib-0017] Hong, C. , Wang, Z. , Zheng, S. L. , Hu, W. J. , Wang, S. N. , Zhao, Y. , & Miao, C. Y. (2023). Metrnl regulates cognitive dysfunction and hippocampal BDNF levels in D‐galactose‐induced aging mice. Acta Pharmacologica Sinica, 44(4), 741–751. 10.1038/s41401-022-01009-y 36229598 PMC10042843

[acel14130-bib-0018] Kasinath, B. S. , Feliers, D. , Sataranatarajan, K. , Ghosh Choudhury, G. , Lee, M. J. , & Mariappan, M. M. (2009). Regulation of mRNA translation in renal physiology and disease. American Journal of Physiology. Renal Physiology, 297(5), F1153–F1165. 10.1152/ajprenal.90748.2008 19535566 PMC2781325

[acel14130-bib-0019] Lee, Y. H. , Park, J. Y. , Lee, H. , Song, E. S. , Kuk, M. U. , Joo, J. , Oh, S. , Kwon, H. W. , Park, J. T. , & Park, S. C. (2021). Targeting mitochondrial metabolism as a strategy to treat senescence. Cell, 10(11), 3003. 10.3390/cells10113003 PMC8616445 34831224

[acel14130-bib-0020] Lesnefsky, E. J. , Chen, Q. , & Hoppel, C. L. (2016). Mitochondrial metabolism in aging heart. Circulation Research, 118(10), 1593–1611. 10.1161/circresaha.116.307505 27174952 PMC5009371

[acel14130-bib-0021] Liu, D. Y. , Thilo, F. , Scholze, A. , Wittstock, A. , Zhao, Z. G. , Harteneck, C. , Zidek, W. , Zhu, Z. M. , & Tepel, M. (2007). Increased store‐operated and 1‐oleoyl‐2‐acetyl‐sn‐glycerol‐induced calcium influx in monocytes is mediated by transient receptor potential canonical channels in human essential hypertension. Journal of Hypertension, 25(4), 799–808. 10.1097/HJH.0b013e32803cae2b 17351372

[acel14130-bib-0022] Lockwich, T. , Pant, J. , Makusky, A. , Jankowska‐Stephens, E. , Kowalak, J. A. , Markey, S. P. , & Ambudkar, I. S. (2008). Analysis of TRPC3‐interacting proteins by tandem mass spectrometry. Journal of Proteome Research, 7(3), 979–989. 10.1021/pr070496k 18205297

[acel14130-bib-0023] Madreiter‐Sokolowski, C. T. , Thomas, C. , & Ristow, M. (2020). Interrelation between ROS and Ca(2+) in aging and age‐related diseases. Redox Biology, 36, 101678. 10.1016/j.redox.2020.101678 32810740 PMC7451758

[acel14130-bib-0024] Miao, J. , Liu, J. , Niu, J. , Zhang, Y. , Shen, W. , Luo, C. , Liu, Y. , Li, C. , Li, H. , Yang, P. , Liu, Y. , Hou, F. F. , & Zhou, L. (2019). Wnt/β‐catenin/RAS signaling mediates age‐related renal fibrosis and is associated with mitochondrial dysfunction. Aging Cell, 18(5), e13004. 10.1111/acel.13004 31318148 PMC6718575

[acel14130-bib-0025] Miguel, V. , Tituaña, J. , Herrero, J. I. , Herrero, L. , Serra, D. , Cuevas, P. , Barbas, C. , Puyol, D. R. , Márquez‐Expósito, L. , Ruiz‐Ortega, M. , Castillo, C. , Sheng, X. , Susztak, K. , Ruiz‐Canela, M. , Salas‐Salvadó, J. , González, M. A. M. , Ortega, S. , Ramos, R. , & Lamas, S. (2021). Renal tubule Cpt1a overexpression protects from kidney fibrosis by restoring mitochondrial homeostasis. The Journal of Clinical Investigation, 131(5), e140695. 10.1172/jci140695 33465052 PMC7919728

[acel14130-bib-0026] Minutolo, R. , Borrelli, S. , & De Nicola, L. (2015). CKD in the elderly: Kidney senescence or blood pressure‐related nephropathy? American Journal of Kidney Diseases, 66(2), 184–186. 10.1053/j.ajkd.2015.05.004 26210723

[acel14130-bib-0027] Müller, M. , Ahumada‐Castro, U. , Sanhueza, M. , Gonzalez‐Billault, C. , Court, F. A. , & Cárdenas, C. (2018). Mitochondria and calcium regulation as basis of neurodegeneration associated with aging. Frontiers in Neuroscience, 12, 470. 10.3389/fnins.2018.00470 30057523 PMC6053519

[acel14130-bib-0028] Nan, J. , Li, J. , Lin, Y. , Saif Ur Rahman, M. , Li, Z. , & Zhu, L. (2021). The interplay between mitochondria and store‐operated Ca(2+) entry: Emerging insights into cardiac diseases. Journal of Cellular and Molecular Medicine, 25(20), 9496–9512. 10.1111/jcmm.16941 34564947 PMC8505841

[acel14130-bib-0029] Oda, S. , Numaga‐Tomita, T. , Kitajima, N. , Toyama, T. , Harada, E. , Shimauchi, T. , Nishimura, A. , Ishikawa, T. , Kumagai, Y. , Birnbaumer, L. , & Nishida, M. (2017). TRPC6 counteracts TRPC3‐Nox2 protein complex leading to attenuation of hyperglycemia‐induced heart failure in mice. Scientific Reports, 7(1), 7511. 10.1038/s41598-017-07903-4 28790356 PMC5548754

[acel14130-bib-0030] O'Sullivan, E. D. , Hughes, J. , & Ferenbach, D. A. (2017). Renal aging: Causes and consequences. Journal of the American Society of Nephrology, 28(2), 407–420. 10.1681/asn.2015121308 28143966 PMC5280008

[acel14130-bib-0031] Shetty, S. , Kumar, R. , & Bharati, S. (2019). Mito‐TEMPO, a mitochondria‐targeted antioxidant, prevents N‐nitrosodiethylamine‐induced hepatocarcinogenesis in mice. Free Radical Biology & Medicine, 136, 76–86. 10.1016/j.freeradbiomed.2019.03.037 30946961

[acel14130-bib-0032] Sturmlechner, I. , Durik, M. , Sieben, C. J. , Baker, D. J. , & van Deursen, J. M. (2017). Cellular senescence in renal ageing and disease. Nature Reviews. Nephrology, 13(2), 77–89. 10.1038/nrneph.2016.183 28029153

[acel14130-bib-0033] Sumbalová, Z. , Uličná, O. , Kucharská, J. , Rausová, Z. , Vančová, O. , Melicherčík, Ľ. , Tvrdík, T. , Nemec, M. , & Kašparová, S. (2022). D‐galactose‐induced aging in rats‐the effect of metformin on bioenergetics of brain, skeletal muscle and liver. Experimental Gerontology, 163, 111770. 10.1016/j.exger.2022.111770 35314269

[acel14130-bib-0034] Ullah, F. , Ali, T. , Ullah, N. , & Kim, M. O. (2015). Caffeine prevents d‐galactose‐induced cognitive deficits, oxidative stress, neuroinflammation and neurodegeneration in the adult rat brain. Neurochemistry International, 90, 114–124. 10.1016/j.neuint.2015.07.001 26209154

[acel14130-bib-0035] Uzhachenko, R. , Boyd, K. , Olivares‐Villagomez, D. , Zhu, Y. , Goodwin, J. S. , Rana, T. , Shanker, A. , Tan, W. J. , Bondar, T. , Medzhitov, R. , & Ivanova, A. V. (2017). Mitochondrial protein Fus1/Tusc2 in premature aging and age‐related pathologies: Critical roles of calcium and energy homeostasis. Aging (Albany NY), 9(3), 627–649. 10.18632/aging.101213 28351997 PMC5391223

[acel14130-bib-0036] Wang, B. , Xiong, S. , Lin, S. , Xia, W. , Li, Q. , Zhao, Z. , Wei, X. , Lu, Z. , Wei, X. , Gao, P. , Liu, D. , & Zhu, Z. (2017). Enhanced mitochondrial transient receptor potential channel, canonical type 3‐mediated calcium handling in the vasculature from hypertensive rats. Journal of the American Heart Association, 6(7), e005812. 10.1161/jaha.117.005812 28711865 PMC5586301

[acel14130-bib-0037] Wang, H. H. , Sun, Y. N. , Qu, T. Q. , Sang, X. Q. , Zhou, L. M. , Li, Y. X. , & Ren, F. Z. (2022). Nobiletin prevents D‐galactose‐induced C2C12 cell aging by improving mitochondrial function. International Journal of Molecular Sciences, 23(19), 11963. 10.3390/ijms231911963 36233264 PMC9569543

[acel14130-bib-0038] Wang, Y. , Wang, Y. , Yang, M. , & Ma, X. (2021). Implication of cellular senescence in the progression of chronic kidney disease and the treatment potencies. Biomedicine & Pharmacotherapy, 135, 111191. 10.1016/j.biopha.2020.111191 33418306

[acel14130-bib-0039] Wu, Y. L. , Xie, J. , An, S. W. , Oliver, N. , Barrezueta, N. X. , Lin, M. H. , Birnbaumer, L. , & Huang, C. L. (2017). Inhibition of TRPC6 channels ameliorates renal fibrosis and contributes to renal protection by soluble klotho. Kidney International, 91(4), 830–841. 10.1016/j.kint.2016.09.039 27979597 PMC5357448

[acel14130-bib-0041] Zhang, J. Q. , Li, Y. Y. , Zhang, X. Y. , Tian, Z. H. , Liu, C. , Wang, S. T. , & Zhang, F. R. (2023). Cellular senescence of renal tubular epithelial cells in renal fibrosis. Frontiers in Endocrinology, 14, 1085605. 10.3389/fendo.2023.1085605 36926022 PMC10011622

