# Supplementary material for: Enhanced TRPC3 transcription through AT1R/PKA/CREB signaling contributes to mitochondrial dysfunction in renal tubular epithelial cells in D‐galactose‐induced accelerated aging mice
Source: Aging Cell. 2024 Feb 28;23(6):e14130. doi: 10.1111/acel.14130 (PMC11166371; doi:10.1111/acel.14130)
Supplement: Supplementary file 1 — Data S1: [file ACEL-23-e14130-s001.docx]

**Supplementary Information**

**METHODS**

**Animals and treatment**

Twelve-week-old male C75/BL6 mice were purchased from SPF Biotechnology (Beijing, China). Twenty-two-month-old (naturally aged) male C75/BL6 mice were purchased from SHULAIBAO Biotech (Wuhan, China). *Trpc3^-/-^* mice were developed at the Comparative Medicine Branch of the National Institute of Environmental Health Sciences (NIEHS) on a 129SvEv/C57BL/6J mixed background as previously described (Hartmann et al., 2008) and the genotypes (wild-type, heterozygote and homozygote) were identified using the methods described in it. These mice were housed in a pathogen-free environment under a 12/12-hour light-dark cycle and with free access to food and water. All animal experiments were performed in compliance with the guidelines approved by the Institutional Animal Care and Use Committee of the Chinese PLA General Hospital. To establish the accelerated aging model, C75/BL6, wild-type, and *Trpc3^-/-^* mice were intervened with subcutaneous injections of D-galactose (G0750, Sigma-Aldrich, St. Louis, MO, USA) at the dose of 150 mg/kg/day for eight weeks (Azman & Zakaria, 2019). C75/BL6 mice were randomly divided into five groups: Control group (n=15), D-gal group (n=15), D-gal plus resveratrol group (n=15, mice were fed with resveratrol by gavage with the dose of 40mg/kg/d for 12 weeks, beginning four weeks before D-gal intervention), D-gal plus losartan group (mice were fed with losartan by gavage with the dose of 10mg/kg/d for 12 weeks, beginning four weeks before D-gal intervention), D-gal plus mitoTEMPO group (mice were intraperitoneally injected with mitoTEMPO with the dose of 2mg/kg/week for 12 weeks, beginning four weeks before D-gal intervention). Losartan, resveratrol and mitoTEMPO were purchased from Selleck.cn (Shanghai, China). After these interventions, mice were euthanized, and tissue samples were isolated for further experiments.

**Cell culture and treatment**

Human renal proximal tubular epithelial cells (HK-2) were purchased from the Cell Bank of the Typical Culture Preservation Committee, Chinese Academy of Sciences (#SCSP-511) and cultured in Dulbecco’s modified Eagle medium/Ham’s F-12 medium (DMEM-F12) (ThermoFisher Scientific, Waltham, MA, USA) containing 10% (v/v) fetal bovine serum (FBS) (Gibco, ThermoFisher), streptomycin (100 μg/mL) and penicillin (100 U/mL) (Beyotime, Shanghai, China). The cells were maintained in a humidified atmosphere in the cell incubator with 5% CO_2_ provided at 37°C. To establish the model of accelerated aging, HK-2 cells were treated with D-gal, combined with other reagents in different settings. H89 (PKA inhibitor) and KG501 (CREB inhibitor) were all purchased from Selleck.

Culture of the primary RTECs were performed as previous described with minor modifications (Li et al., 2022). Briefly, the kidneys cortical parts of these mice were minced, and subsequently digested in pre-warmed 0.2% (wt/vol) collagenase II solution at 37°C for 30 minutes with frequent mixing, after which the digested tissue was sieved successively through two sieves (pore size 180 μm and 100 μm), then the tubular fragments were resuspended in DMEM-F12 medium supplemented with 10% FBS with both streptomycin and penicillin. Cells were cultured for 4-7 days until they reached 60% to 80% confluency. Cells of the second and third passages were used for subsequent experiments.

**Small Interfering RNA (siRNA) and cell transfections**

An effective siRNA for CREB was purchase from Santa Cruz Biotechnology (#sc-29281, Santa Cruz, CA, USA). CREB overexpression plasmid was constructed by GeneCopoeia (Guangzhou, China) and confirmed by plasmid sequencing. Cell transfections were performed according to previous study. Lipofectamine 3000 and Opti-MEM were all purchased from Invitrogen (Carlsbad, CA, USA).

**Renal pathology and immunohistochemistry**

Mice kidneys were fixed with 10% formalin (Shanghai Acmec Biochemical, Shanghai, China), embedded in paraffin (Acmec) and sectioned to 4 μm thickness, with H&E staining (G1120, Solarbio Life Sciences, Beijing, China), PAS staining (G1281, Solarbio) and Masson trichrome staining (G1346, Solarbio) according to the manufacturers’ protocols (Miao et al., 2019). Images were visualized and acquired using an Olympus BX 53 microscope, while the PAS-positive and the fibrotic area were quantified by the Image J software (Rasband, W.S., U. S. National Institutes of Health).

As to immunohistochemistry, paraffin sections were subjected to dewaxing, rehydration and antigen retrieval. Then the sections were blocked with 5% BSA， incubated with primary antibodies (1:100) for an overnight at 4°C, then washed with PBS, and incubated with appropriate secondary antibodies in 5% BSA solution for 1 h at room temperature (25°C). For a negative control, the primary antibody was omitted with no staining occurred. The DAB (3,3'-diaminobenzidine)-positive area or DAB-positive nuclei percentage was quantified by the Image J software. Primary antibodies included: anti-AT1R (#ab124505; Abcam, Cambridge, MA, USA), anti-Sirt1 (#8469; Cell signaling technology, Danvers, MA, USA), anti-P16^INK4a^ (#ab252788; Abcam), anti-Collagen I (#ab270993, Abcam), anti-Collagen IV (#ab6586; Abcam), anti-Fibronectin (#ab2413; Abcam) and TRPC3 (#ACC-016, Alomone Labs, Jerusalem, Israel).

**Biochemical tests**

The serum levels of urea nitrogen and creatinine were measured using QuantiChrom Urea Assay Kit (DIUR-500, BioAssay Systems, Hayward, CA, USA) and QuantiChrom Creatinine Assay Kit (DICT-500, BioAssay Systems), followed by the manufacturer’s instructions. Urinary albumin and urinary creatinine levels were measured using commercial kits (Jiancheng Bioengineering, Nanjing, China) according to the manufacturer’s instructions.

**Western blotting**

Protein levels were examined by western blotting according to a previous study (Wang et al., 2017). In brief, kidney cortical tissues and HK-2 cells were lysed with RIPA buffer (Beyotime) supplemented with cocktail of protease inhibitors (Roche, Mannheim, Germany). The lysates were then centrifuged at 12,000×*g* for 15 minutes at 4 °C and the supernatants were collected, with protein concentrations determined with BCA kit (Beyotime). Proteins were separated with a 10% SDS-PAGE gel and transferred to a PVDF membrane (Merck Millipore, Massachusetts, USA). After blocking with 5% BSA, membranes were incubated with primary antibodies and corresponding HRP-conjugated secondary antibodies (Beyotime). The bands were then visualized using a chemiluminescence HRP substrate (Merck Millipore) and quantified using the Image J software. Primary antibodies used were as follows: anti-Collagen I (#ab270993, Abcam), anti-Collagen IV (#31882; Cell Signaling Technology), anti-Fibronectin (#ab2413; Abcam), anti-glyceraldehyde 3-phosphate dehydrogenase (GAPDH, #AF0006, Beyotime), anti-Sirt1 (#8469; Cell Signaling Technology), anti-P16^INK4A^ (#29271; Cell Signaling Technology), anti-P21 (#ab188224; Abcam), anti-TRPC1/3/6/7 (#ACC-010, #ACC-016, #ACC-017, #ACC-043; Alomone Labs, Jerusalem, Israel), anti-AT1R (#ab124505; Abcam), anti-phosphorylated PKA and total PKA (#5661, #4782; Cell Signaling Technology), anti-phosphorylated CREB, total CREB (#9198, #9197; Cell Signaling Technology), interleukin-6 (#DF6087; Affinity Biosciences, Jiangsu, China), MCP-1 (#DF7577; Affinity) and TGF-β1 (#ab215715; Abcam).

**Mitochondrial function assays**

Mitochondrial respiratory functions were analyzed using Oxygraph-2k respirometer (Oroboros Instruments, Innsbruck, Austria) according to previous reports (Gao et al., 2020; Wang et al., 2017). In general, both HK-2 cells and primary RTECs were harvested and tested by adding a series of substrates and inhibitors of different complexes located at the mitochondrial respiratory chain. Routine OCR, CI_OXPHOS_, CI+II_OXPHOS_, and CI+II_ETS_ values were measured and calculated.

Enzyme activities of mitochondrial respiratory chain complexes were measured using commercial kits from Abcam (#ab109721 and #ab109908) according to the protocols provided. The protein concentrations were measured using the BCA kit (Beyotime). Enzyme activities were presented as the catalytic rate (mOD/min/mg protein).

Measurement of ATP synthesis was conducted using a firefly luciferase-based ATP assay kit (Beyotime) according to the provided protocols. ATP contents were presented as arbitrary units normalized to the control group.

Measurement of the MMP was performed using a JC-1 kit from Beyotime. HK-2 cells and primary RTECs were incubated with JC-1 dyes for 30 minutes. The changes of MMP values were presented with the ratio of red fluorescence intensity (detected with excitation/emission wavelengths of 525/590 nm) over green fluorescence intensity (detected with excitation/emission wavelengths of 490/530 nm). Fold changes over the control group were presented.

Measurement of the ROS levels was detected using a dihydroethidium (DHE) fluorescent probe for cytosolic ROS and a MitoSOX Red dye (ThermoFisher Scientific, Waltham, MA) for mitochondrial ROS in both primary RTECs and HK-2 cells. The detailed protocols were adopted as previous reported (Wang et al., 2017). The quantification of ROS levels was conducted using a Fluoroskan Ascent Fluorometer (ThermoFisher, Helsinki, Finland) with the excitation wavelength 510nm and the emission wavelength 580nm.

**Quantitative reverse transcription-polymerase chain reaction (QRT-PCR)**

Hk-2 cells and kidney cortical tissues were lysed with TRIzol reagent (Invitrogen). The quality and concentrations of total RNAs were measured using a NanoDrop spectrophotometer (Wilmington, Delaware). The mRNA levels of TRPC channels were measured using Premix Ex Taq (Takara, Kusatsu, Japan) in LightCycler System (Roche Diagnostics). The TRPCs mRNA levels were normalized to the relative expression of GAPDH using the 2^−ΔΔCt^ method. The primers were synthesized by Invitrogen and the sequence of the primers were listed in Table S1.

**Intracellular and mitochondrial calcium patterns measurements**

Evaluation of the intracellular and mitochondrial calcium patterns was conducted using the Fura-2 AM and Rhod-2 AM dyes (ThermoFisher Scientific, Waltham, MA) according to a previous study (Wang et al., 2017). For intracellular calcium measurement, primary RTECs were resuspended in Hank’s Balance Salt Solution (HBSS) and incubated with Fura-2 AM dye. OAG (membrane permeant diacylglycerol analog) was used to activate calcium influx via TRPCs, while TG was used to inhibit endoplasmic reticulum Ca^2+^-ATPase thus inducing SOCE. SOCEs inhibitor 2-APB, TRPCs inhibitor SKF-96365 and TRPC3 inhibitor Pyr3 (all from Sigma-Aldrich) were used to further investigate the role of TRPC channels in RTECs. F340nm/F380nm ratio was used to reflect the changes of [Ca^2+^]_cyto_ in each experiment.

For mitochondrial calcium measurement, cells were loaded with Rhod-2 AM, while agonists (ATP, from Sigma-Aldrich) and calcium were added to detect the fluorescence intensity at an emission wavelength of 581 nm plus an excitation wavelength of 552 nm, both at baseline and after treatment. R_X_/R_0_ ratios were used to reflect the changes of [Ca^2+^]_mito_.

**Dual-luciferase reporter assay**

The whole-length promoter region of the TRPC3 gene was identified in the UCSC Genome Browser website (http://genome.ucsc.edu/). JASPAR database (http://jaspar.genereg.net/) was used to predict the potential biding sites of CREB in the TRPC3 promoter region sequence. The plasmids used for luciferase reporter assay were constructed by incorporating the fragments of the promoter regions PT1 (-2300 to +401), PT2 (-1832 to +401), PT3 (-1520 to +401), into the pGL3.0 vector. PT1-Mut and PT2-Mut plasmids were constructed by mutation of the CRE binding points and then cloned into the pGL3.0 vector. The primers used were provided in Table S1.

Dual luciferase reporter assay was performed according to a previous report (Hu et al., 2015). In brief, HK-2 cells were co-transfected with the above-mentioned pGL3.0-based recombinant plasmids and pRL-TK (Promega Corporation, Wisconsin, USA) using Lipofectamine 3000 (Invitrogen). Dual-luciferase reporter assays (#E1960, Promega) were conducted based on the manufacturer’s instructions at 36 hours after co-transfection. The ratio of firefly to Renilla luciferase activity was calculated as the relative luciferase activity. **ChIP assay**

ChIP assay was performed using the Pierce™ agarose ChIP kit (ThermoFisher Scientific) according to the provided protocols. In brief, HK-2 cells were treated with KG-501 or vehicle, then the cells were treated with 1% formaldehyde to cross-link chromatin-associated proteins to DNA. The cell lysates were isolated and sonicated to shear the DNA to an average length between 200 to 1000 bp (SONIC, Newtown, CT, USA). Subsequently, the chromatin was immunoprecipitated with antibodies against CREB (#9197, ChIP grade, Cell Signaling Technology), with equal amounts of IgG (#2729, Cell Signaling Technology) as a negative control, with 20 μL magnetic protein G beads overnight at 4 °C with slow rotation. Then, qPCR was performed to amplify the fragments covering CRE1, CRE2 and an intron in the TRPC3 promoter region sequence with triplicates. The sequences of the primers used were also listed in Table S1.

**Statistically analysis**

All the data were presented as the mean ± standard deviation (mean ± SD). The statistical analysis was performed using the one-way ANOVA followed by Bonferroni's multiple comparisons test. A two-tailed *P* <0.05 was regarded as statistically significant.

**References**

Azman, K. F., & Zakaria, R. (2019). D-Galactose-induced accelerated aging model: an overview. *Biogerontology, 20*(6), 763-782. doi:10.1007/s10522-019-09837-y

Gao, P., Jiang, Y., Wu, H., Sun, F., Li, Y., He, H., Wang, B., Lu, Z., Hu, Y., Wei, X., Cui, Y., He, C., Wang, L., Zheng, H., Yang, G., Liu, D., Yan, Z., & Zhu, Z. (2020). Inhibition of Mitochondrial Calcium Overload by SIRT3 Prevents Obesity- or Age-Related Whitening of Brown Adipose Tissue. *Diabetes, 69*(2), 165-180. doi:10.2337/db19-0526

Hartmann, J., Dragicevic, E., Adelsberger, H., Henning, H. A., Sumser, M., Abramowitz, J., Blum, R., Dietrich, A., Freichel, M., Flockerzi, V., Birnbaumer, L., & Konnerth, A. (2008). TRPC3 channels are required for synaptic transmission and motor coordination. *Neuron, 59*(3), 392-398. doi:10.1016/j.neuron.2008.06.009

Hu, C. J., Wang, B., Tang, B., Chen, B. J., Xiao, Y. F., Qin, Y., Yong, X., Luo, G., Zhang, J. W., Zhang, D., Li, S., He, F., & Yang, S. M. (2015). The FOXM1-induced resistance to oxaliplatin is partially mediated by its novel target gene Mcl-1 in gastric cancer cells. *Biochim Biophys Acta, 1849*(3), 290-299. doi:10.1016/j.bbagrm.2014.11.008

Li, X. T., Song, J. W., Zhang, Z. Z., Zhang, M. W., Liang, L. R., Miao, R., Liu, Y., Chen, Y. H., Liu, X. Y., & Zhong, J. C. (2022). Sirtuin 7 mitigates renal ferroptosis, fibrosis and injury in hypertensive mice by facilitating the KLF15/Nrf2 signaling. *Free Radic Biol Med, 193*(Pt 1), 459-473. doi:10.1016/j.freeradbiomed.2022.10.320

Miao, J., Liu, J., Niu, J., Zhang, Y., Shen, W., Luo, C., Liu, Y., Li, C., Li, H., Yang, P., Liu, Y., Hou, F. F., & Zhou, L. (2019). Wnt/β-catenin/RAS signaling mediates age-related renal fibrosis and is associated with mitochondrial dysfunction. *Aging Cell, 18*(5), e13004. doi:10.1111/acel.13004

Wang, B., Xiong, S., Lin, S., Xia, W., Li, Q., Zhao, Z., Wei, X., Lu, Z., Wei, X., Gao, P., Liu, D., & Zhu, Z. (2017). Enhanced Mitochondrial Transient Receptor Potential Channel, Canonical Type 3-Mediated Calcium Handling in the Vasculature From Hypertensive Rats. *J Am Heart Assoc, 6*(7), e005812. doi:10.1161/jaha.117.005812

**SUPPLEMENTAL FIGURES**
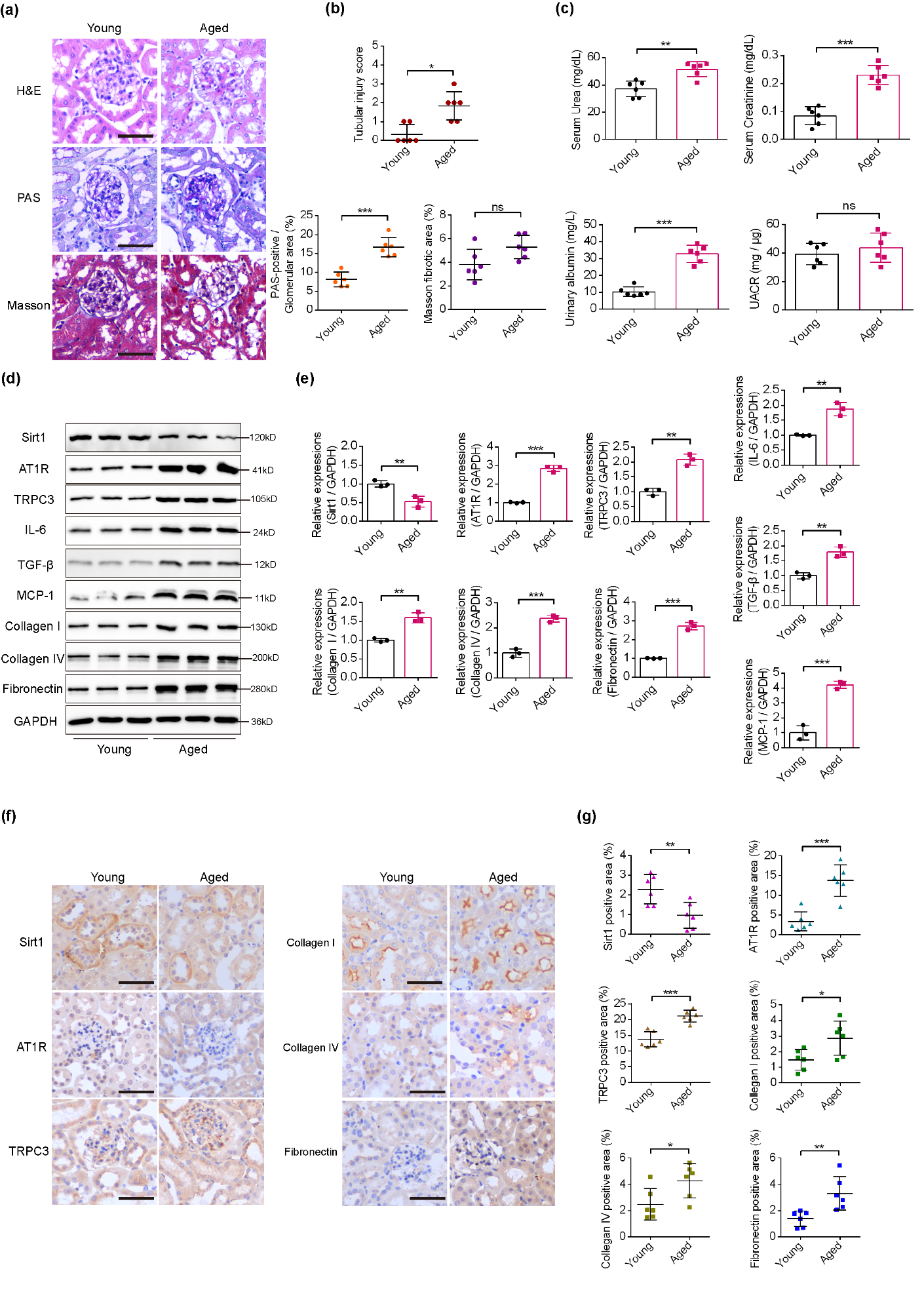


**FIGURE S1**. Kidney changes of naturally-aged mice compared with young mice. (a, b) Representative kidney sections of young (6-month old) and aged (22-month old) mice, stained with H&E, PAS and Masson dyes, with tubular injury score analysis, PAS-positive area percentage and Masson fibrotic area percentage presented (n=6). (c) The urinary albumin concentrations (mg/L) and urinary albumin/creatinine ratio (mg/μg) levels in both young and aged mice (n=6). (d, e) Immunoblotting evaluation of protein levels of senescence-associated (Sirt1, AT1R, TRPC3), senescence-associated secretory phenotype (IL-6, TGF-β, MCP-1) and fibrotic markers (Collagen I, Collagen IV, Fibronectin) in renal tissues, with GAPDH as a loading control in the above-mentioned mice. Quantitative analysis of these bands was performed and calculated for statistical significance (n=3). (f, g) Representative kidney sections of the above-mentioned mice, with immunohistochemical staining of Sirt1, AT1R, TRPC3, Collagen I, Collagen IV and Fibronectin. Positive area percentage of these target proteins were analyzed by Image J software (n=6). **P*<0.05, ***P*<0.01, ****P*<0.001.


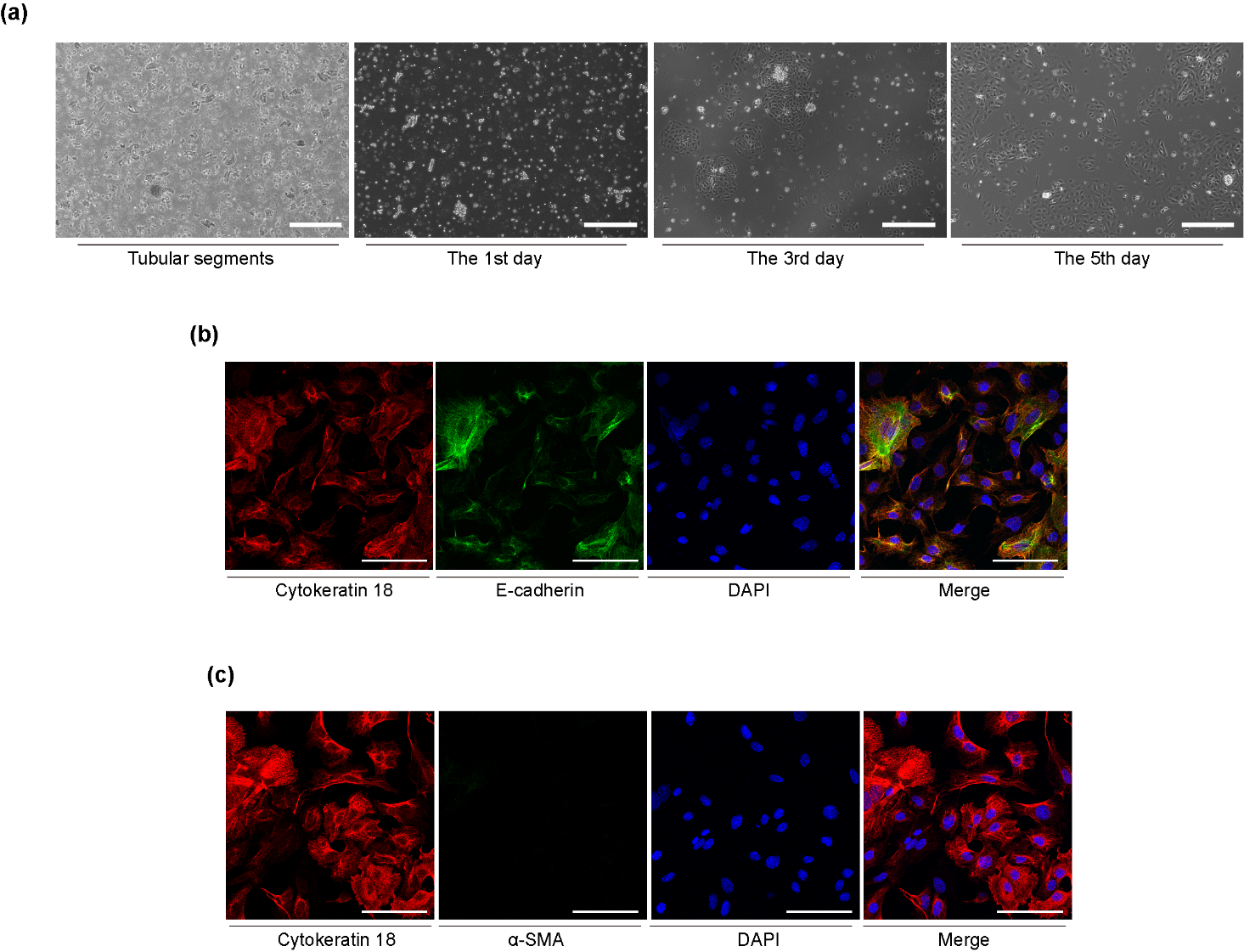


**FIGURE S2.** Culture and identification of primary renal tubular cells (RTECs). (a) The process of isolation and culture of primary RTECs using mice kidney tissues. (b) Representative images of dual immunofluorescence staining confirmed cytokeratin 18 (red) and E-cadherin (green) expression in primary mouse RTECs. (c) Representative images of dual immunofluorescence staining confirmed cytokeratin 18 (red) and α-SMA (green) expression in primary mouse RTECs.

**Table S1** The primers used in this study.

| Gene name | Forward sequence | Reverse sequence |
| --- | --- | --- |
| TRPC1 | 5'-TGGATTATTGGGATGATTTGG-3' | 5'-CACTTTGAGGGCAAAGGTTG-3' |
| TRPC3 | 5'-GGTTAAACCTCTTCACACAGTCTAA-3' | 5'-TTGTGCTTTCAAAACATACCG-3' |
| TRPC6 | 5'-TTGCAAGATTTATGGCGTTC-3' | 5'-TCCTGGCCAGATTGTAGTATTT-3' |
| TRPC7 | 5'-CACCAGATACCAGAAGATCATGAAG-3' | 5'-AGCCTGAGACTTCTCCTCCA-3' |
| GAPDH | 5'-GGCAAATTCAACGGCACAGT-3' | 5'-CGCTCCTGGAAGATGGTGAT-3' |
| PT1 | 5’-CGAGCCTTTTGAGATCAGAAACTTCTCC-3’ | 5’-CTTCCTGACCTTGGTGGACAGAAC-3’ |
| PT2 | 5’-CGAGCCATGGTCCTATAAGGGCAGTTT-3’ | 5’-CTTCCTGACCTTGGTGGACAGAAC-3’ |
| PT3 | 5’-CGAGAAGAGGGCTGTCAGACTGGA-3’ | 5’-CTTCCTGACCTTGGTGGACAGAAC-3’ |
| ChIP Primer-1 | 5’- GTTGTTTAAGCCACCC-3’ | 5’-TATAGGACCATGGATAGAAG-3’ |
| ChIP Primer-2 | 5’-TCTAAATAGCAAGGACAAAA-3’ | 5’-CTGTTTCCTCTAAACTATGA-3’ |
| ChIP Primer-3 | 5’-GCTCTGTCCAGGCTCAA-3’ | 5’-TCCTCGGCTCTAATACAGG-3’ |
